# Mitochondria-targeted cuproptosis-driven nanoplatform for synergistic photothermal/chemodynamic therapy and systemic antitumor immunotherapy

**DOI:** 10.1016/j.mtbio.2026.103303

**Published:** 2026-05-30

**Authors:** Hui Zhang, Yuting Lu, Jingchun Wang, Yikai Ma, Yuanjin Sun, Shengzhong Rong, Xu Zhu, Yingxue Jin

**Affiliations:** a College of Public Health, Mudanjiang Medical University, Mudanjiang, 157009, China; b Key Laboratory for Photonic and Electronic Bandgap Materials, Ministry of Education, College of Chemistry & Chemical Engineering, Harbin Normal University, Harbin, 150025, China; c Heilongjiang Nursing College, Harbin, 150025, China

**Keywords:** Cuproptosis, Mitochondrial targeting, Tumor microenvironment remodeling, Cancer immunotherapy, Carbon dots

## Abstract

Cuproptosis, a recently identified copper-dependent regulated cell death pathway driven by mitochondrial lipoylated protein aggregation, holds considerable promise for cancer therapy. Its clinical translation is constrained by off-target toxicity of free copper ions, inadequate mitochondria-targeted delivery and the immunosuppressive tumor microenvironment. We developed a hyaluronic acid-camouflaged, pH-responsive nanoplatform HACCR for mitochondria-targeted, cuproptosis-driven synergistic therapy and systemic antitumor immunity. The nanoplatform core relies on self-assembled architecture via π-π stacking between copper-metformin carbon dots and immune adjuvant R837, achieving high payload efficiencies 43.6% for CuMCDs and 35.2% for R837. CuMCDs synthesized via one-pot hydrothermal synthesis exhibit intrinsic mitochondria-targeting properties, facilitating precise copper delivery to cuproptosis initiation sites and amplifying pathway activation. They display efficient photothermal conversion under 808 nm laser irradiation, directly inducing tumor cell death while enhancing cuproptosis, chemodynamic therapy and immunogenic cell death. The hyaluronic acid-cinnamaldehyde micelle shell enables CD44-mediated tumor targeting and acidic tumor microenvironment-responsive payload release to mitigate off-target effects. In vitro and *in vivo* evaluations confirm effective cuproptosis induction, immunosuppressive tumor microenvironment reversal and enhanced dendritic cell maturation and T cell infiltration. Combined with anti-PD-L1 checkpoint blockade, HACCR achieves robust primary tumor eradication and distant metastasis inhibition with favorable systemic biosafety. This work establishes a versatile nanoplatform for cuproptosis-based synergistic cancer immunotherapy, offering insights for clinical translation of copper-dependent cell death strategies.

## Highlights

• A mitochondria-targeted, pH-responsive nanoplatform (HACCR) is rationally designed for cuproptosis-driven synergistic cancer immunotherapy.
• Carrier-free CuMCDs@R837 nanoassemblies achieve ultrahigh drug loading efficiency, avoiding carrier-related systemic toxicity and metabolic risks.
• The inherent mitochondria-targeting capability of CuMCDs enables precise copper delivery to the cuproptosis core site, drastically amplifying the cuproptosis effect.
• The all-in-one nanoplatform integrates PTT, CDT, cuproptosis and immunotherapy, with photothermal effect further boosting antitumor efficacy and ICD induction.
• HACCR effectively remodels the immunosuppressive TME, and combined with anti-PD-L1 blockade, achieves complete primary tumor eradication and robust distant metastasis inhibition.

## Graphical abstract

**Figure:**
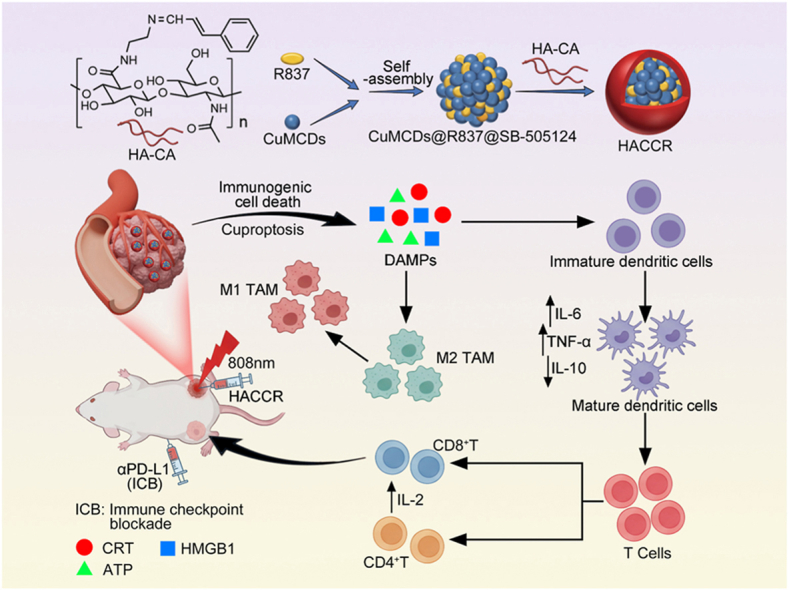

## 1 Introduction

Malignant tumors remain a severe threat to global public health, and conventional therapies including chemotherapy and radiotherapy are greatly restricted by systemic side effects, drug resistance and limited control over tumor metastasis and recurrence [1,2]. Regulated cell death pathways with distinct mechanisms have provided new directions for the development of effective cancer treatment strategies [3,4]. Cuproptosis was identified as a novel copper-dependent regulated cell death manner in 2022 [5]. This death pattern is mediated by the aggregation of mitochondrial lipoylated proteins involved in the tricarboxylic acid cycle and subsequent proteotoxic stress, which is independent of caspase activation and thus provides a promising strategy to overcome apoptosis resistance in tumors [[6], [7], [8], [9], [10], [11]].

However, the clinical translation of cuproptosis-based therapy is hampered by several critical limitations. Free copper ions bring severe off-target toxicity and poor tumor accumulation [12]. The inefficient mitochondrial delivery of copper ions restricts the activation of cuproptosis and reduces therapeutic efficacy [13,14]. High-level glutathione in the tumor microenvironment chelates copper ions and scavenges reactive oxygen species to weaken cuproptosis and chemodynamic therapy effects [15,16]. Hypoxic and immunosuppressive tumor microenvironment further limits systemic antitumor immune responses and impedes the inhibition of metastatic tumors [17,18].

Nanomedicine promotes tumor-targeted accumulation and subcellular delivery of therapeutic agents [19]. Traditional nanocarriers are limited by low drug loading efficiency, complex preparation and carrier-related toxicity or metabolic risks [20]. Carbon dots have attracted extensive attention in biomedical fields owing to good biocompatibility, facile synthesis, tunable optical properties and versatile surface modification [[21], [22], [23]]. Metformin shows inherent mitochondrial targeting ability, and such characteristic can be inherited by metformin-derived carbon dots during preparation [24].

Given that cuproptosis mainly occurs in mitochondria, metformin is used as a precursor to construct copper-doped carbon dots with inherent mitochondrial targeting capacity to achieve targeted cuproptosis induction and enhanced antitumor efficiency.

A mitochondria-targeted and pH-responsive nanoplatform HACCR is constructed for cuproptosis-driven synergistic photothermal therapy, chemodynamic therapy and systemic antitumor immunotherapy. Copper-metformin carbon dots synthesized via one-pot hydrothermal reaction act as a copper donor for cuproptosis and possess inherent mitochondrial targeting, near-infrared photothermal conversion and fluorescence imaging properties. Carrier-free nanoassemblies are formed through π-π stacking between copper-metformin carbon dots and the TLR7 agonist R837 to realize high drug loading without exogenous carriers. The nanoassemblies are further encapsulated into pH-responsive micelles composed of hyaluronic acid-cinnamaldehyde conjugates linked by Schiff base bonds. The hyaluronic acid shell mediates CD44-dependent tumor targeting, and acid-labile Schiff base bonds enable rapid drug release in acidic tumor microenvironments and lysosomes.

The carrier-free design based on copper-metformin carbon dots ensures high drug loading efficiency and avoids carrier-associated risks. Inherent mitochondrial targeting ability delivers copper ions to the core site of cuproptosis, improves cuproptosis induction efficiency and reduces off-target toxicity of free copper ions. The integrated nanoplatform combines tumor targeting, pH-responsive release, photothermal therapy, chemodynamic therapy, cuproptosis and immunotherapy. Photothermal effects directly kill tumor cells and amplify cuproptosis, chemodynamic therapy and immunogenic cell death to achieve synergistic antitumor effects. The nanoplatform depletes intracellular glutathione and generates oxygen to alleviate tumor hypoxia and downregulate the HIF-1α/VEGF/PD-L1 pathway. Immunogenic cell death cooperates with R837 to promote dendritic cell maturation, induce M2-to-M1 macrophage polarization and enhance cytotoxic T cell infiltration to reshape the immunosuppressive tumor microenvironment. Combined with *anti*-PD-L1 immune checkpoint blockade, the nanoplatform triggers robust systemic antitumor immune responses and inhibits the growth of distant metastatic tumors. The intrinsic fluorescence of copper-metformin carbon dots allows real-time imaging tracking at cellular and *in vivo* levels, and photothermal imaging supports precise photothermal therapy to realize imaging-guided theranostics.

**Scheme 1:**
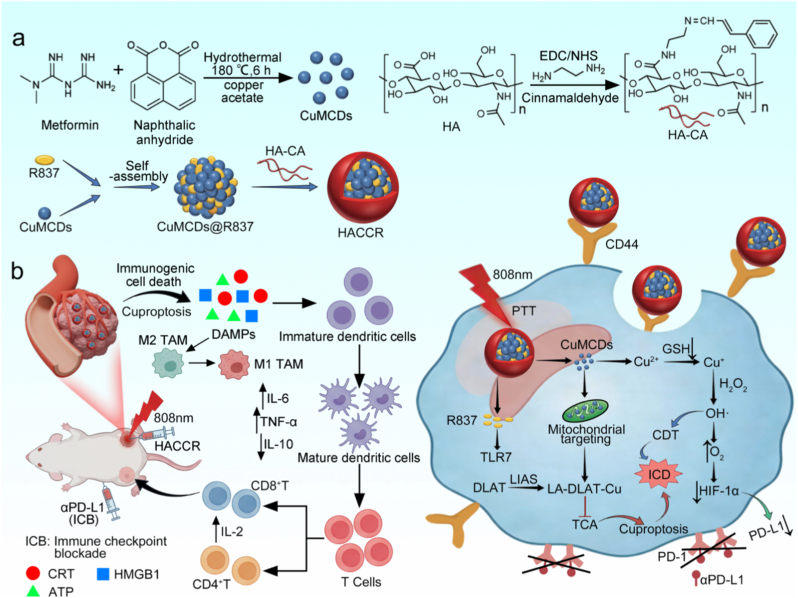
Schematic illustration of the synthesis procedure of the HACCR nanoplatform and the underlying mechanism of cuproptosis-driven synergistic antitumor therapy and systemic immune activation.

HACCR induces obvious glutathione depletion and reactive oxygen species accumulation, which are similar to the features of ferroptosis and disulfidptosis. Systematic mechanistic verification confirms that HACCR-mediated cell death mainly depends on cuproptosis rather than ferroptosis or disulfidptosis. The absence of iron and the lack of necessary structural motifs for disulfidptosis activation exclude the two death pathways. The copper-dependent cell death shows typical cuproptosis characteristics including the aggregation of mitochondrial lipoylated proteins, which is independent of ferroptotic and disulfidptotic pathways. Glutathione depletion and reactive oxygen species production act as synergistic regulators to reduce copper chelation and enhance oxidative stress for amplified cuproptosis efficiency. The synthesis and antitumor mechanism of HACCR are illustrated in Scheme 1.

## 2 Results and discussion

### 2.1 Design and synthesis of the HACCR nanoplatform

The synthetic pathway of HACCR and the corresponding mechanism of cuproptosis-driven synergistic antitumor immunotherapy are presented in Scheme 1. CuMCDs were prepared through a one-pot hydrothermal treatment of copper acetate, metformin and 1,8-naphthalic anhydride under controlled temperature and duration [25]. CuMCDs and R837 were then assembled through π–π stacking interactions to form carrier-free CuMCDs@R837 nanostructures. Hyaluronic acid was modified with cinnamaldehyde through amide condensation to generate pH-responsive HA-CA polymers with Schiff base linkages. These polymers further assembled into micellar structures to encapsulate CuMCDs@R837 and form the final HACCR nanoplatform.

Systemic administration allows HACCR to accumulate in tumor tissues through CD44-mediated active targeting and the enhanced permeability and retention effect [26]. The acidic tumor microenvironment induces the cleavage of Schiff base structures within HA-CA micelles [27]. Such structural dissociation triggers the disassembly of the micellar shell and the release of the CuMCDs@R837 core. Internalized CuMCDs distribute preferentially to mitochondria and release copper ions to promote the aggregation of lipoylated proteins involved in the tricarboxylic acid cycle. This process further initiates strong cuproptotic responses in tumor cells.

CuMCDs consume intracellular glutathione to elevate oxidative stress levels and catalyze the production of hydroxyl radicals through Fenton-like reactions to support chemodynamic therapy. Photothermal effects derived from CuMCDs under 808 nm laser irradiation lead to direct tumor cell ablation and reinforce cuproptosis, chemodynamic therapy and immunogenic cell death. Damage-associated molecular patterns released from dying tumor cells act in concert with R837 to facilitate dendritic cell maturation and antigen presentation. These events drive the phenotypic transformation of tumor-associated macrophages from immunosuppressive M2 to pro-inflammatory M1 subtypes and activate cytotoxic T lymphocyte-mediated systemic antitumor immune responses. Combined administration with anti-PD-L1 immune checkpoint blockade enhances the suppression of both primary and distant metastatic tumor lesions.

### 2.2 Physicochemical characterization of the nanoplatform

**Fig. 1:**
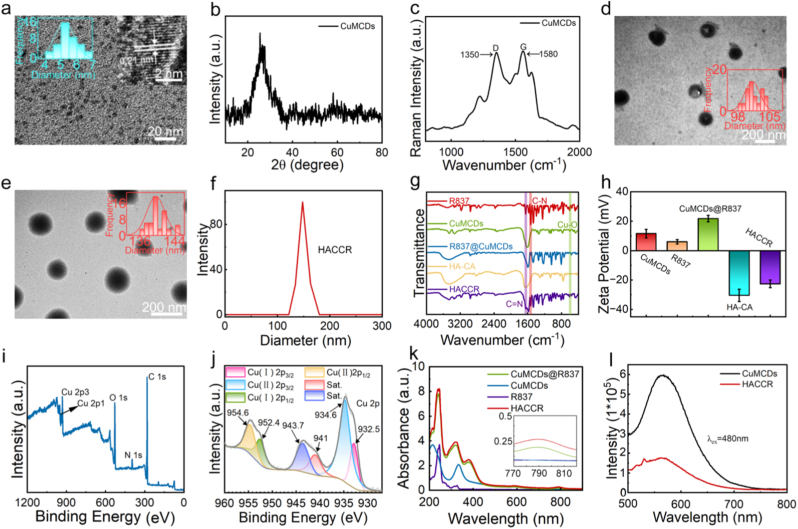
Physicochemical characterizations of CuMCDs, CuMCDs@R837 nanocomposites, and HACCR nanoplatform. (a) TEM image of CuMCDs; inset: size distribution and HRTEM image. (b) XRD pattern of CuMCDs. (c) Raman spectrum of CuMCDs. (d) TEM image of CuMCDs@R837; inset: size distribution. (e) TEM image of HACCR; inset: size distribution. (f) DLS size profile of HACCR. (g) FT-IR spectra of the samples. (h) Zeta potentials of the samples. (i) XPS survey spectrum of CuMCDs. (j) High-resolution Cu 2p XPS spectrum of CuMCDs. (k) UV-vis-NIR absorption spectra of the samples; inset: magnified view of the 770–810 nm region. (l) Fluorescence emission spectra of CuMCDs and HACCR (λ_ex_ = 480 nm).

Transmission electron microscopy was applied to characterize the morphology and structural features of the prepared nanomaterials (Fig. 1a). The synthesized CuMCDs presented uniform spherical shapes and favorable monodispersity. Statistical size distribution indicates an average diameter of approximately 5 nm. High-resolution transmission electron microscopy images reveal the typical amorphous structure of carbon nanodots. The X-ray diffraction pattern of CuMCDs shows a broad diffraction peak centered at about 25°, which corresponds to the amorphous carbon framework and supports the successful preparation of carbon dots (Fig. 1b). Raman spectra (Fig. 1c) exhibit two characteristic bands located at approximately 1350 cm^−1^ and 1580 cm^−1^. These signals are assigned to the D band related to disordered carbon structures and the G band related to graphitic sp^2^ carbon domains, respectively. The spectral features match well with the structural properties of carbon dot-based materials.

Assembly with R837 yields CuMCDs@R837 nanoparticles that retain satisfactory monodispersity with increased particle dimensions (Fig. 1d). Encapsulation within HA-CA micelles results in the formation of HACCR nanostructures with distinct core-shell configurations. The average particle size is measured at approximately 140 nm, which falls within the suitable range for tumor accumulation through the enhanced permeability and retention effect (Fig. 1e). Dynamic light scattering measurements provide hydrodynamic size information consistent with transmission electron microscopy observations (Fig. 1f). Fourier transform infrared spectroscopy was used to confirm the formation of key chemical structures (Fig. 1g). The spectrum of HA-CA shows a characteristic absorption signal of C=N Schiff base structures at approximately 1640 cm^−1^. This result verifies the successful conjugation of cinnamaldehyde onto the hyaluronic acid backbone through Schiff base linkage. Characteristic absorption features of R837 and CuMCDs are preserved in the spectrum of HACCR, which indicates the effective encapsulation of the CuMCDs@R837 core. Zeta potential (Fig. 1h) monitoring during the sequential assembly process provides additional evidence for structural construction. Pure CuMCDs exhibit positive surface potential values. The CuMCDs@R837 assembly formed through π–π stacking interactions maintains positive potential characteristics, which excludes electrostatic adsorption as the main driving force for the assembly process. The positively charged core is then encapsulated by negatively charged HA-CA polymers through electrostatic interactions. The final HACCR nanostructures show negative surface potential, which helps reduce non-specific protein adsorption and extend blood circulation duration *in vivo*.

X-ray photoelectron spectroscopy was used to analyze the elemental composition and chemical valence states of CuMCDs (Fig. 1i and j). Full survey spectra confirm the presence of carbon, nitrogen, oxygen and copper elements. High-resolution Cu 2p spectra reveal characteristic peaks of Cu(I) and Cu(II) species. The coexistence of these two copper valence states provides a stable copper source for cuproptosis induction and supports Fenton-like reactions for chemodynamic therapy.

Optical properties were evaluated using ultraviolet-visible-near infrared absorption and fluorescence spectroscopy (Fig. 1k and l). CuMCDs show broad absorption in the near infrared region with obvious absorption at 808 nm, which provides the basis for effective near infrared laser-induced photothermal therapy. The near infrared absorption characteristics are retained after assembly and encapsulation processes. Fluorescence emission spectra indicate strong emission of CuMCDs under 480 nm excitation. The fluorescence intensity of HACCR is significantly reduced due to aggregation-induced quenching effects. Excited state energy is more inclined to dissipate in the form of thermal energy rather than fluorescence emission, which is consistent with the efficient photothermal conversion performance of the nanoplatform.

Energy dispersive X-ray spectroscopy elemental mapping was used to characterize the elemental distribution of HACCR. Carbon, nitrogen, oxygen and copper elements are evenly distributed throughout the nanostructures. This result confirms the uniform encapsulation of CuMCDs within the nanoplatform.

### 2.3 pH-responsive performance and colloidal stability

Schiff base linkages incorporated in the HA-CA assembly enable structural destabilization under acidic conditions that resemble the lysosomal lumen or tumor extracellular microenvironment. Incubation of HACCR under physiological pH preserves the structural integrity of the core-shell architecture for an extended duration. Exposure to acidic conditions promotes gradual disassembly of the micellar framework in a time-dependent manner (Fig. S2a). The accelerated release of CuMCDs and R837 occurs under acidic conditions while release is suppressed at neutral pH (Fig. S2b and c). Such a profile favors retention of the payload during systemic circulation and supports controlled liberation within pathological sites to minimize unintended interactions with healthy tissues.

Suspensions of HACCR were maintained in saline, neutral buffer and complete cell culture medium supplemented with serum over one week to assess colloidal stability. Hydrodynamic size, optical absorbance and surface charge remain unchanged throughout the monitoring period (Fig. S3). The unchanged physicochemical properties confirm the structural robustness of the nanoplatform under conditions that mimic physiological environments. Stable colloidal behavior supports prolonged retention in the circulatory system and helps maintain consistent biological performance after administration.

The design assumes acid-labile behavior arises exclusively from Schiff base hydrolysis. Structural destabilization could also arise from non-specific erosion or serum-induced destabilization that is unrelated to pH responsiveness. The interpretation of release profiles as tumor-specific activation ignores potential premature disassembly in inflamed healthy tissues with moderately acidic pH. Colloidal stability was only assessed in static aqueous environments without considering shear forces or protein coronas that alter nanomaterial behavior *in vivo*.

### 2.4 Photothermal performance and ROS-related functions

**Fig. 2:**
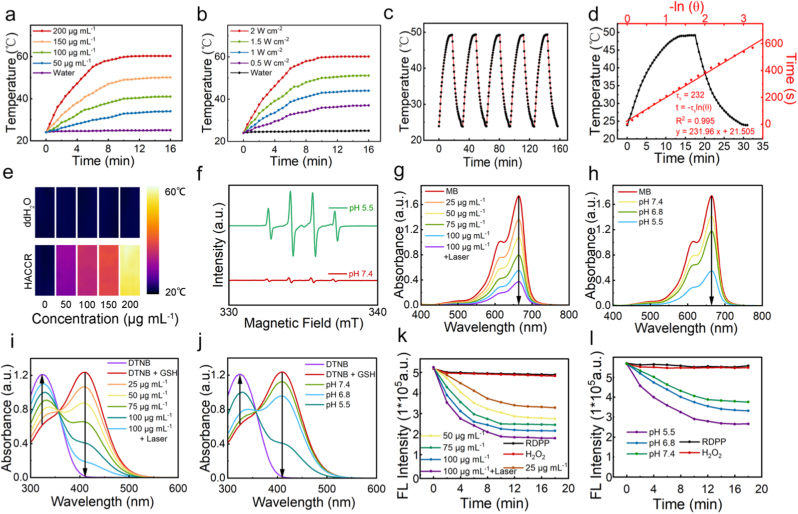
Functional evaluations of the HACCR nanoplatform. (a) Photothermal heating curves of HACCR at different concentrations under 808 nm laser irradiation. (b) Photothermal heating curves at different laser power densities. (c) Photothermal cycling stability. (d) Photothermal conversion efficiency calculation. (e) Infrared thermal images at different concentrations with water as the control. (f) ESR spectra of •OH at different pH values. (g, h) Methylene blue (MB) degradation profiles at different concentrations and pH conditions. (i, j) Glutathione (GSH) depletion efficiency at different concentrations and pH values. (k, l) Oxygen (O_2_) generation capacity at different concentrations and pH values. Data are presented as mean ± SD (n = 3). Statistical differences were determined by one-way ANOVA.

Irradiation with 808 nm laser illumination induces temperature elevation in aqueous suspensions of HACCR. Temperature change scales with nanomaterial concentration and laser power density (Fig. 2a and b). Repeated heating and cooling cycles do not compromise photothermal performance (Fig. 2c). The photothermal conversion efficiency reaches 38.2% and exceeds values reported for many carbon-based photothermal agents (Fig. 2d). Infrared thermal imaging confirms spatially localized heating that correlates with quantitative temperature measurements (Fig. 2e).

Electron spin resonance spectroscopy confirms the formation of hydroxyl radicals under acidic conditions. Neutral conditions fail to support detectable radical generation (Fig. 2f). Methylene blue degradation proceeds more efficiently at lower pH and higher HACCR concentrations (Fig. 2g and h). These observations support a pH-dependent activation profile for Fenton-like catalytic activity. Incubation with HACCR reduces the concentration of glutathione in a concentration-dependent and pH-dependent manner (Fig. 2i and j). Depletion of intracellular glutathione weakens antioxidant defense systems and reduces copper chelation capacity. Reduced glutathione availability amplifies oxidative stress and enhances the availability of copper species to promote downstream biological effects.

Hypoxia within the tumor microenvironment compromises therapeutic efficacy and supports immune escape mechanisms [28,29]. Treatment with HACCR increases oxygen availability in a concentration-dependent manner with enhanced activity under acidic conditions (Fig. 2k and l). Elevated oxygen tension alleviates hypoxic stress and modulates downstream signaling pathways related to angiogenesis and immune suppression [30,31]. Endogenous hydrogen peroxide levels in the tumor microenvironment provide sufficient substrate for oxygen generation without the need for exogenous supplementation.

Photothermal efficacy is demonstrated in idealized aqueous conditions without accounting for light scattering and absorption by biological tissues that reduce heating efficiency *in vivo*. Hydroxyl radical detection does not exclude other reactive oxygen species that may contribute to biological effects. The contribution of heat-induced glutathione oxidation versus chemical-mediated depletion remains uncharacterized. Oxygen generation was measured using fluorescent probes rather than direct quantification, and sustained oxygen production over time was not evaluated. The conclusion that modulation of HIF-1α, VEGF and PD-L1 arises directly from oxygen generation ignores potential contributions from photothermal damage or copper-mediated signaling. The assumption that endogenous hydrogen peroxide is universally sufficient across tumor types neglects heterogeneity in metabolic profiles that may limit catalytic activity.

### 2.5 *In vitro* tumor cell targeting and functional evaluation

**Fig. 3:**
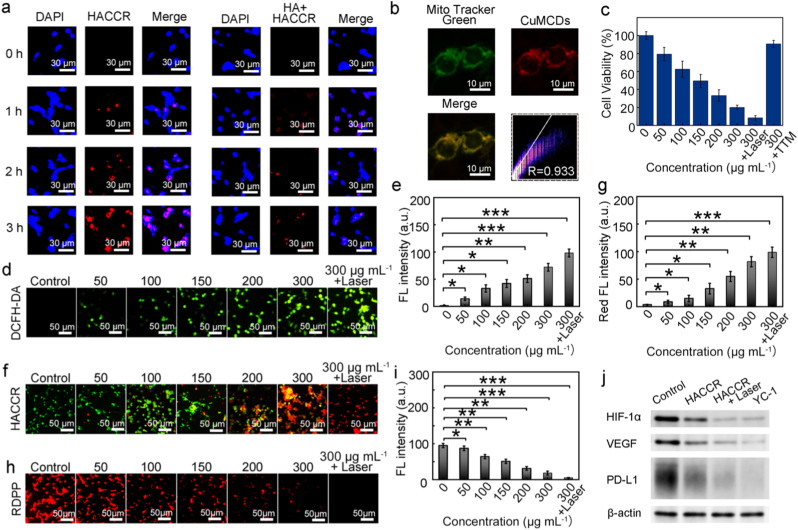
In vitro cellular targeting and functional validation of HACCR. (a) Time-dependent cellular uptake of HACCR in CT26 cells and uptake inhibition via CD44 blocking with free HA. (b) Mitochondrial colocalization analysis of HACCR. (c) Relative viability of CT26 cells under different treatments. (d,e) Intracellular ROS imaging and quantitative analysis. (f,g) Live/dead staining and fluorescence quantification of CT26 cells. (h,i) Intracellular oxygen level imaging and quantification for hypoxia relief. (j) Western blot analysis of HIF-1α, VEGF and PD-L1 expression; YC-1 (1-(5-Chloroisobenzofuranyl-1H-pyrazole), a specific HIF-1α inhibitor), was used as the positive control. Data are presented as mean ± SD (n = 3). Statistical differences were determined by one-way ANOVA. *∗P* < 0.05, ∗∗*P* < 0.01, ∗∗∗*P* < 0.001. Fluorescence quantification was performed using ImageJ.

Cellular internalization profiles of HACCR were investigated in CD44-overexpressing CT26 cells. Confocal laser scanning microscopy observations presented in Fig. 3a illustrate the gradual elevation of intracellular fluorescent signals derived from HACCR with prolonged incubation periods. Excess free HA administered prior to HACCR treatment suppresses the internalization process. These observations validate the contribution of CD44-mediated recognition mechanisms to the selective uptake of HACCR by tumor cells.

Mitochondrial accumulation of CuMCDs was assessed through colocalization analysis. Visualization in Fig. 3b demonstrates substantial overlap between fluorescent signals of HACCR and mitochondrial probes with a Pearson correlation coefficient of 0.933. Efficient mitochondrial deposition facilitates the targeted delivery of copper ions to subcellular sites closely associated with cuproptosis initiation. The mitochondrial tropism of CuMCDs arises from multiple structural characteristics. Metformin incorporated as a synthetic precursor may preserve biological recognition properties after integration into carbon nanostructures. Positively charged surface properties promote electrostatic interactions with negatively charged mitochondrial compartments. Nanoscale dimensions around 5 nm support penetration of intracellular barriers to access mitochondrial microenvironments. The combined effects enable specific mitochondrial localization without additional chemical modifications.

Cell viability monitoring reveals concentration-dependent cytotoxicity of HACCR against CT26 cells as depicted in Fig. 3c. Laser irradiation further potentiates the cytotoxic effects. Pretreatment with tetrathiomolybdate, a copper-specific chelator, effectively abolishes the cytotoxicity induced by HACCR with laser irradiation. These results indicate the essential contribution of copper-associated processes to the therapeutic activity. Therapeutic efficacy of HACCR exceeds that of copper sulfate at equivalent copper concentrations as shown in Fig. S4. The HACCR with laser treatment achieves substantial cell elimination at defined concentrations while copper sulfate produces limited cytotoxic effects. Mitochondrial targeting capacities improve copper utilization efficiency and enhance cuproptosis induction compared with freely diffusing copper ions. Live/dead staining assays presented in Fig. 3f and g confirm the progressive elevation of non-viable cell populations with increasing HACCR concentrations. Laser treatment results in extensive cell death at comparable concentrations. Copper sulfate treatments produce minimal cytotoxic effects as illustrated in Fig. S5. These findings support the advantages of the nanoscale delivery system for copper-mediated therapeutic applications.

Intracellular reactive oxygen species levels were monitored using DCFH-DA probes. Quantitative analysis in Fig. 3d and e demonstrates concentration-dependent elevation of fluorescent signals with further enhancement under laser irradiation. Increased oxidative stress contributes to chemodynamic therapeutic activity in conjunction with glutathione depletion mechanisms. Modulation of intracellular hypoxic status was evaluated using oxygen-sensitive probes. Quantitative analysis in Fig. 3h and i illustrates concentration-dependent increases in intracellular oxygen levels following HACCR administration. Western blot (WB) analysis in Fig. 3j confirms downregulated expression of hypoxia-inducible factor-1α and its downstream effectors vascular endothelial growth factor and programmed death ligand-1. Laser irradiation enhances these regulatory effects. Alleviated hypoxic stress contributes to the modulation of immunosuppressive tumor microenvironments and improved therapeutic responsiveness.

### 2.6 Mechanism verification of HACCR-induced cuproptosis

**Fig. 4:**
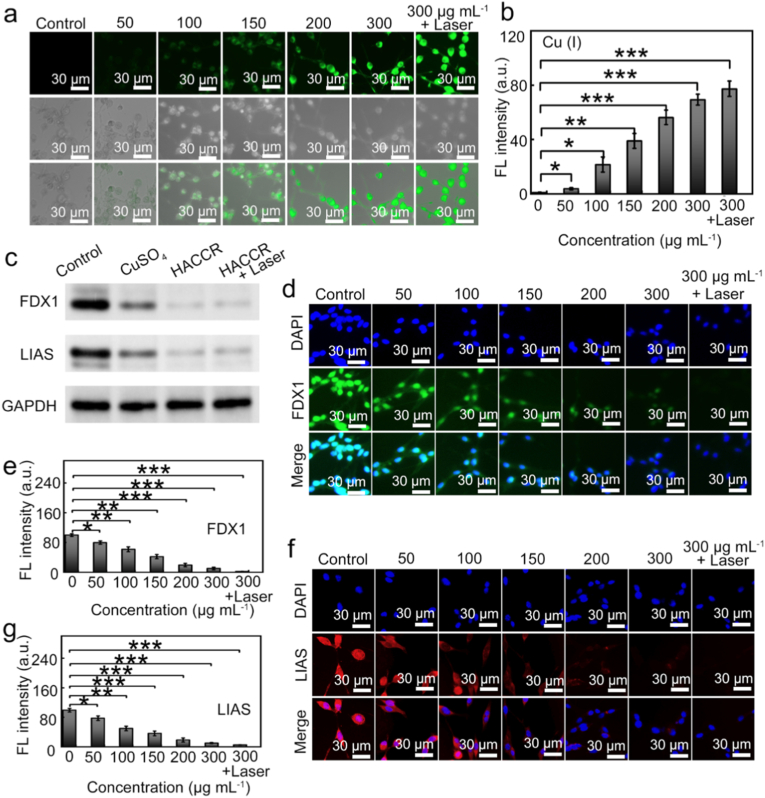
Validation of HACCR-induced cuproptosis in CT26 cells. (a, b) Intracellular Cu(I) imaging and semi-quantitative analysis at different HACCR concentrations. (c) Western blot analysis of cuproptosis-associated proteins FDX1 and LIAS. (d, e) Immunofluorescence imaging and semi-quantification of FDX1. (f, g) Immunofluorescence imaging and semi-quantification of LIAS. Data are presented as mean ± SD (n = 3). Statistical differences were determined by one-way ANOVA. ∗*P* < 0.05, ∗∗*P* < 0.01, ∗∗∗*P* < 0.001. Fluorescence quantification was performed using ImageJ.

Intracellular Cu (I) concentrations were monitored to assess the initiation of copper-associated signaling events. Fluorescence imaging and quantitative analysis presented in Fig. 4a and b reveal concentration-dependent elevation of Cu (I) levels following HACCR administration. Laser irradiation further enhances the accumulation of Cu (I) species to provide sufficient copper availability for downstream biological responses. Expression profiles of cuproptosis-associated regulatory proteins were evaluated through western blot analysis. Data presented in Fig. 4c demonstrate significant downregulation of ferredoxin 1 and lipoic acid synthase expression following HACCR treatment. Laser irradiation strengthens the regulatory effects on these protein targets. Free copper sulfate produces relatively weak modulation of these proteins compared with HACCR treatment. Immunofluorescence imaging confirms concentration-dependent reduction of ferredoxin 1 and lipoic acid synthase signals as shown in Fig. 4d, e, 4f and 4g. Pretreatment with tetrathiomolybdate abolishes the regulatory effects on these protein targets as illustrated in Figs. S6 and S7. These observations confirm copper-dependent modulation of cuproptosis-associated protein expression.

HACCR triggers copper-dependent cell death through the regulation of cuproptosis-associated proteins and mitochondrial function. The absence of iron components and structural motifs required for disulfidptosis excludes these death pathways from contributing to observed biological effects. Copper chelation effectively reverses protein expression changes and mitochondrial dysfunction to confirm copper dependency in observed cellular responses. Mitochondrial targeting capabilities facilitate the specific delivery of copper ions to subcellular compartments associated with cuproptosis activation. Characteristic aggregation patterns of dihydrolipoamide acetyltransferase polypeptide further support the activation of cuproptosis signaling. Glutathione depletion and reactive oxygen species accumulation act as synergistic modulators to enhance copper-dependent cellular responses rather than initiating alternative death pathways.

Photothermal effects reinforce cuproptosis, chemodynamic therapy and immunogenic cell death through complementary mechanisms. Thermal stimulation promotes the structural dissociation of CuMCDs to facilitate copper release. Elevated temperatures enhance mitochondrial membrane permeability to promote interactions between copper species and lipoylated mitochondrial proteins. Photothermal effects accelerate glutathione depletion to strengthen chemodynamic therapeutic activity and promote cellular stress responses that facilitate immunogenic cell death. These complementary mechanisms contribute to enhanced therapeutic outcomes through coordinated biological regulation.

**Fig. 5:**
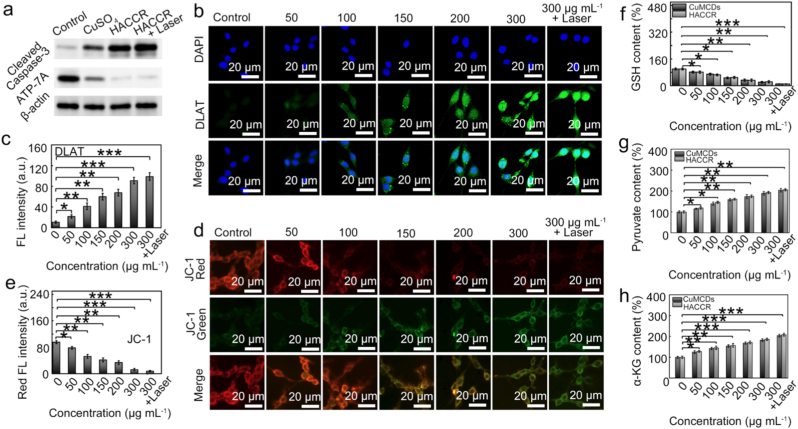
Downstream mechanistic validation of HACCR-elicited cuproptosis in CT26 cells. (a) WB detection of cleaved Caspase-3 and ATP7A expression under different treatments. (b, c) Immunofluorescence imaging and semi-quantitative analysis of cuproptosis-associated DLAT at gradient HACCR concentrations. (d, e) JC-1 staining and red fluorescence semi-quantification for evaluating mitochondrial membrane potential. (f–h) Detection of intracellular GSH, pyruvate and α-ketoglutarate (α-KG) levels upon HACCR treatment. Data are presented as mean ± SD (n = 3). Statistical significance was analyzed by one-way ANOVA. ∗*P* < 0.05, ∗∗*P* < 0.01, ∗∗∗*P* < 0.001. Fluorescence quantification was performed using ImageJ.

Activation of downstream cuproptosis signaling and mitochondrial functional perturbation was evaluated in HACCR-treated CT26 cells. WB analysis presented in Fig. 5a shows elevated levels of cleaved Caspase-3 in cells exposed to HACCR. This change arises as a secondary consequence of extensive mitochondrial damage and oxidative stress rather than direct activation of conventional apoptotic cascades. Expression of the copper transporter ATP7A is simultaneously reduced. These observations support the predominance of cuproptosis over conventional apoptosis in HACCR-mediated cell death [[32], [33], [34]]. Immunofluorescence imaging illustrates concentration-dependent enhancement of DLAT expression and aggregation in cells treated with HACCR as shown in Fig. 5b and c. Laser irradiation further promotes these molecular changes. Free copper sulfate produces limited effects on DLAT while tetrathiomolybdate pretreatment effectively reverses copper-mediated DLAT alterations as presented in Fig. S8. These findings confirm the benefits of mitochondrial copper targeting in amplifying cuproptosis-related molecular events.

Mitochondrial membrane potential was monitored using JC-1 probes to evaluate functional integrity. Data presented in Fig. 5d and e demonstrate concentration-dependent reduction of mitochondrial membrane potential following HACCR treatment. This disruption manifests as diminished red fluorescence and enhanced green fluorescence signals. Severe mitochondrial impairment further promotes cuproptosis progression as mitochondria represent the primary platform for cuproptosis execution [[35], [36], [37]]. Tetrathiomolybdate pretreatment preserves mitochondrial membrane potential to confirm copper-dependent dysfunction as illustrated in Fig. S9.

Intracellular metabolic profiles associated with cuproptosis were quantitatively assessed. HACCR treatment reduces intracellular glutathione content in a concentration-dependent manner as shown in Fig. 5f. This observation aligns with previously evaluated glutathione depletion profiles. Levels of pyruvate and α-ketoglutarate, key metabolites in the tricarboxylic acid cycle, are significantly elevated following HACCR administration as presented in Fig. 5g and h. Such metabolic disturbances represent characteristic features of cuproptosis-associated tricarboxylic acid cycle dysregulation. These metabolic changes provide additional evidence supporting HACCR-mediated cuproptosis induction at the metabolic level.

### 2.7 *In vitro* ICD induction and immune cell regulation

**Fig. 6:**
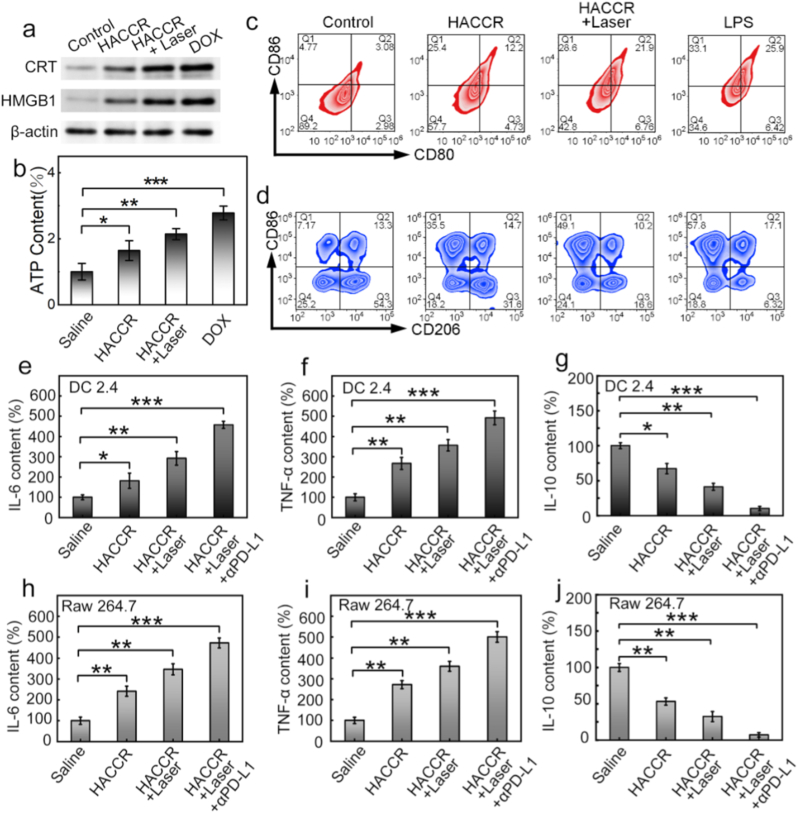
Assessment of HACCR-triggered ICD and immune modulation. (a) Western blot analysis of ICD-associated CRT and HMGB1 expression under various treatments. (b) Extracellular ATP secretion from CT26 cells in different treatment groups. (c) Flow cytometry analysis of CD80/CD86 maturation markers in DC2.4 cells co-cultured with treated CT26 cells. (d) Flow cytometry detection of CD86/CD206 polarization markers in Raw264.7 macrophages after co-culture. (e–g) Levels of pro-inflammatory IL-6, TNF-α and anti-inflammatory IL-10 in DC2.4 cells. (h–j) Secretion profiles of IL-6, TNF-α and IL-10 in Raw264.7 macrophages. Data are presented as mean ± SD (n = 3). Statistical significance was analyzed by one-way ANOVA. ∗*P* < 0.05, ∗∗*P* < 0.01, ∗∗∗*P* < 0.001. Fluorescence quantification was performed using ImageJ.

Immunogenic cell death converts immunosuppressive tumor cells into immunostimulatory entities through the release of damage associated molecular patterns to initiate antitumor immune responses [[38], [39], [40]]. Western blot analysis presented in Fig. 6a shows that HACCR treatment enhances the expression of calreticulin and high mobility group box 1, two representative damage associated molecular patterns. Laser irradiation further strengthens the upregulation of these markers. Extracellular adenosine triphosphate levels are also elevated following HACCR treatment with additional enhancement under laser irradiation as illustrated in Fig. 6b. These observations confirm the ability of HACCR mediated cuproptosis combined with photothermal and chemodynamic therapy to trigger immunogenic cell death in tumor cells.

Modulation of dendritic cell maturation was examined through co-culture systems. Flow cytometric analysis reveals the expression profiles of CD80 and CD86 in DC2.4 cells following co-incubation with treated CT26 cells [41,42]. Data presented in Fig. 6c demonstrate significantly increased proportions of mature dendritic cells in groups treated with HACCR and laser irradiation. The observed maturation efficiency exceeds that of lipopolysaccharide positive control groups. The combined effects of immunogenic cell death and R837 adjuvant activity contribute to efficient dendritic cell maturation which initiates adaptive immune responses against tumor entities.

Phenotypic modulation of tumor associated macrophages was evaluated to assess tumor microenvironment remodeling. Flow cytometric analysis presented in Fig. 6d shows increased expression of CD86 and decreased expression of CD206 in Raw264.7 macrophages following co-incubation with treated CT26 cells [43]. These observations indicate the transformation of tumor associated macrophages from immunosuppressive M2 toward proinflammatory M1 phenotypes to alleviate immunosuppressive conditions within the tumor microenvironment.

Cytokine secretion profiles were evaluated to assess immune activation states. Levels of interleukin 6 and tumor necrosis factor α are elevated in DC2.4 cells while interleukin 10 levels are reduced following HACCR treatment as shown in Fig. 6e–g. Laser irradiation and anti-PD-L1 combination further enhance these regulatory effects. Similar cytokine profiles are observed in Raw264.7 macrophages with increased proinflammatory factor secretion and decreased anti-inflammatory factor production as illustrated in Fig. 6h–j. These results confirm the ability of HACCR to activate proinflammatory functions of innate immune cells and establish favorable conditions for subsequent adaptive immune activation.

### 2.8 *In vivo* tumor targeting and photothermal performance

*In vivo* fluorescence imaging was applied to assess the tumor accumulation of HACCR in CT26 tumor-bearing mice. Fluorescence signals at tumor sites increase progressively after intravenous administration and reach maximum intensity at 8 h as shown in Fig. S10a and S10b. Observable signals persist through 48 h to support effective tumor retention. The combined effects of CD44-mediated recognition and enhanced permeability and retention effect contribute to the selective tumor accumulation. Ex vivo imaging of major organs and tumor tissues at 48 h confirms significantly higher fluorescence intensity in tumors than in healthy tissues as presented in Fig. S10c and S10d. Such selective distribution helps reduce unintended exposure to normal organs and limit potential off-target effects.

In vivo photothermal performance was evaluated using infrared thermal imaging. Temperature at tumor sites increases rapidly during laser irradiation at various time points after administration. Maximum temperature elevation occurs at 8 h, which coincides with the period of highest tumor accumulation as illustrated in Fig. S10e and S10f. These observations confirm the suitability of HACCR for effective photothermal therapy in physiological environments.

### 2.9 *In vivo* antitumor efficacy evaluation

**Fig. 7:**
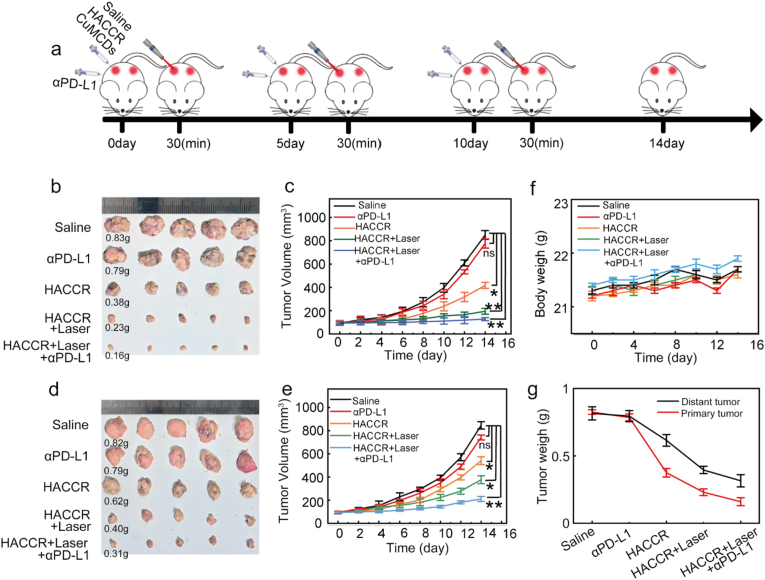
*In vivo* anti-tumor efficacy of HACCR combined with PTT and ICB. (a) Schematic of administration and laser irradiation protocol in bilateral CT26 tumor-bearing mice. (b) Representative images of primary tumors post-treatment. (c) Primary tumor growth curves during treatment. (d) Representative images of distant tumors post-treatment. (e) Distant tumor growth curves during treatment. (f) Mouse body weight changes during treatment. (g) Primary and distant tumor weight statistics post-treatment. Data are presented as mean ± SD (n = 5). Statistical significance was analyzed by one-way ANOVA. ∗*P* < 0.05, ∗∗*P* < 0.01, ∗∗∗*P* < 0.001.

Therapeutic efficacy was assessed in bilateral CT26 tumor models that simulate both primary and distant lesions. Mice were randomized into groups receiving saline, αPD-L1, HACCR, HACCR with laser irradiation or combined HACCR, laser and αPD-L1. Therapeutic agents were administered at designated intervals with laser treatments performed shortly after administration as depicted in Fig. 7a.

Primary tumor growth profiles show rapid expansion in saline and αPD-L1 groups with limited therapeutic benefit as presented in Fig. 7b and c. HACCR treatment produces moderate tumor suppression while laser irradiation enhances therapeutic outcomes. The triple combination regimen results in substantial regression of primary tumors with the highest therapeutic efficiency. Distant tumors without direct laser irradiation exhibit similar growth patterns to primary lesions. Significant growth suppression occurs only in the triple combination group as shown in Fig. 7d and e. These findings indicate the establishment of systemic antitumor immunity that controls both local and distant lesions beyond localized photothermal effects. Quantitative analysis confirms higher inhibition rates in the triple combination group compared with all other treatments. Body weight remains stable throughout the experimental period without significant fluctuations across all groups as illustrated in Fig. 7f. These results indicate favorable biocompatibility and minimal systemic toxicity. Tumor weight measurements at the experimental endpoint are consistent with growth profiles and further validate the therapeutic benefits of combination regimens as presented in Fig. 7g. In the primary tumor model, the monotherapy with αPD-L1 yielded only 4.82% tumor growth inhibition relative to the saline control. In contrast, treatment with HACCR alone, HACCR plus laser irradiation, and the triple combination of HACCR, laser, and αPD-L1 achieved inhibition rates of 54.22%, 72.29%, and 80.72%, respectively. In the distant tumor model, corresponding inhibition rates were 3.66% (αPD-L1 monotherapy), 24.39% (HACCR alone), 51.22% (HACCR plus laser), and 62.20% (the triple combination). Statistical analysis confirmed that the triple therapy significantly outperformed all other regimens in suppressing both primary and distant tumors (∗*P* < 0.05).

Certain limitations exist in the current experimental design. The absence of a laser plus αPD-L1 control group prevents definitive evaluation of potential interactions between photothermal effects and immune checkpoint blockade. The lack of free CuMCDs and R837 monotherapy groups limits the differentiation of individual contributions from each functional component. Future investigations will incorporate these control groups to clarify the specific roles and synergistic mechanisms of each therapeutic element.

### 2.10 *In vivo* biosafety evaluation

Systemic biosafety profiles were assessed through histopathological examination hematological analysis and metabolic tracking of key components. Histological evaluation of major organs reveals intact tissue architecture without evidence of inflammation necrosis or structural abnormalities across all treatment groups as shown in Fig. S11. These observations confirm favorable organ compatibility following administration of the therapeutic formulations.

Neurological safety was specifically evaluated considering the potential for copper species to penetrate biological barriers. Brain tissue sections maintain normal cytoarchitecture without neuronal damage or inflammatory infiltration as illustrated in Fig. S12. No evidence of neurotoxicity is detected in treated animals compared with control groups. Tumor tissue sections demonstrate extensive necrosis nuclear condensation and lysis in groups receiving laser irradiation and combination treatments. These pathological changes confirm effective tumor growth suppression while sparing healthy tissues. Blood biochemistry and hematological parameters remain within normal physiological ranges across all experimental groups as presented in Fig. S13. Liver and renal function indicators show no significant abnormalities. Hematological profiles maintain stable populations of cellular components. These results confirm the absence of overt organ toxicity or systemic disturbances following treatment. Liver clearance of copper components was quantified through inductively coupled plasma mass spectrometry. Fecal copper concentrations show progressive elimination over time as summarized in Table S1. Most copper species are excreted within several days after administration. Efficient metabolic clearance reduces potential long term accumulation of metal components and minimizes associated safety risks.

### 2.11 *In vivo* immune regulation mechanism

**Fig. 8:**
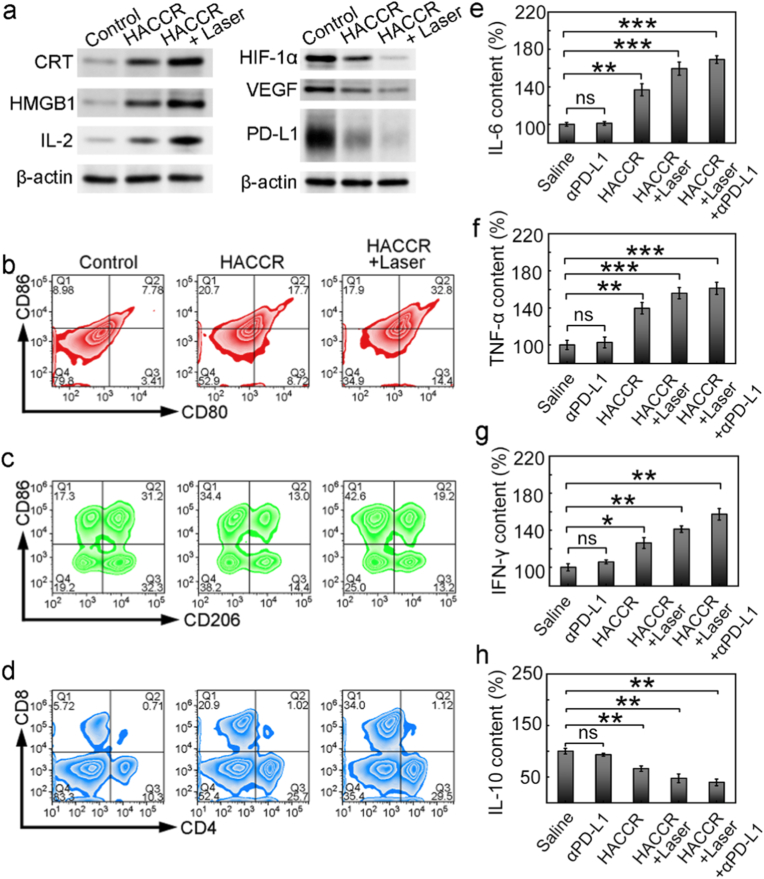
In vivo immune regulatory mechanism of HACCR-mediated synergistic therapy. (a) Western blot analysis of CRT, HMGB1, IL-2, HIF-1α, VEGF and PD-L1 in tumor tissues. (b) Flow cytometric analysis of CD80 and CD86 in dendritic cells from tumor-draining lymph nodes. (c) Flow cytometric analysis of CD86 and CD206 for macrophage polarization in tumor tissues. (d) Flow cytometric analysis of CD4^+^ and CD8^+^ T lymphocyte subsets in tumor tissues. (e-h) Levels of IL-6, TNF-α, IFN-γ and IL-10 in tumor tissues. Data are presented as mean ± SD (n = 5). Statistical differences were determined by one-way ANOVA. ∗*P* < 0.05, ∗∗*P* < 0.01, ∗∗∗*P* < 0.001.

Immunomodulatory effects of HACCR-mediated combinatorial therapy were investigated through molecular and cellular profiling in tumor tissues and draining lymph nodes. Western blot analysis of tumor homogenates demonstrates enhanced expression of CRT and HMGB1 alongside elevated IL-2 levels in mice receiving HACCR with laser irradiation as shown in Fig. 8a. The expression of HIF-1α, VEGF and PD-L1 is simultaneously reduced. These observations confirm the induction of immunogenic cell death and suppression of hypoxia-linked immunosuppressive signaling in the tumor microenvironment.

Flow cytometric analysis of tumor-draining lymph nodes reveals increased proportions of mature dendritic cells in animals treated with HACCR and laser irradiation as illustrated in Fig. 8b. Enhanced dendritic cell maturation facilitates effective tumor antigen presentation and supports the initiation of adaptive immune responses. Immune cell infiltration profiles within tumor tissues demonstrate phenotypic conversion of tumor-associated macrophages following HACCR treatment. Increased M1-type macrophage proportions coincide with reduced M2-type populations as presented in Fig. 8c. Combination therapy including HACCR, laser irradiation and αPD-L1 further promotes the infiltration of CD4^+^ and CD8^+^ T lymphocytes into tumor parenchyma as shown in Fig. 8d. Enhanced effector T cell accumulation reinforces tumor-specific cytotoxic immune responses.

Cytokine profiling of tumor tissues confirms the establishment of pro-inflammatory microenvironmental conditions. Levels of IL-6, TNF-α and IFN-γ are significantly elevated while IL-10 expression is reduced in the combination therapy group as depicted in Fig. 8e–h. The coordinated modulation of cytokine networks establishes a favorable immune microenvironment that supports systemic antitumorimmunity.

## 3 Conclusion

A pH-responsive and mitochondria-targeted theranostic nanoplatform denoted as HACCR is developed for cuproptosis-mediated synergistic cancer therapy. The carrier-free assembly based on copper-metformin carbon dots supports high payload integration and reduces risks associated with traditional vector systems. Intrinsic mitochondria-targeting properties facilitate the precise deposition of copper ions at subcellular sites closely related to cuproptosis activation. The multifunctional system combines tumor-specific accumulation, stimuli-responsive payload release, photothermal conversion, chemodynamic activity and immune regulatory capacity. Photothermal effects generated by copper-metformin carbon dots reinforce cuproptosis activation, chemodynamic performance and immunogenic cell death induction. Systematic evaluations confirm the efficient induction of copper-dependent cell death in tumor compartments. The nanoplatform remodels immunosuppressive tumor microenvironments and promotes the activation and infiltration of immune effector cells. When combined with immune checkpoint blockade, the system achieves robust control over both primary lesions and distant tumor growth while maintaining favorable biosafety profiles. This work establishes an integrated strategy for copper-dependent cell death-based synergistic cancer treatment. The platform expands the development of stimulus-responsive nanomedicines for tumor theranostics and provides insights for the clinical translation of cuproptosis-mediated therapeutic approaches.

## 4 Experimental section

### 4.1 Materials and reagents

Copper acetate, metformin hydrochloride, 1,8-naphthalic anhydride, hyaluronic acid (HA, MW ∼10 kDa), cinnamaldehyde (CA), 1-(3-Dimethylaminopropyl)-3-ethylcarbodiimide hydrochloride (EDC), N-Hydroxysuccinimide (NHS), R837, and tetrathiomolybdate (TTM) were purchased from Aladdin Biochemical Technology Co., Ltd. (Shanghai, China). 2′,7′-Dichlorodihydrofluorescein diacetate (DCFH-DA), MitoTracker Green, JC-1 probe, Calcein-AM/PI live/dead staining kit, DAPI, and BCA protein assay kit were purchased from Beyotime Biotechnology Co., Ltd. (Shanghai, China). Primary antibodies against CRT, HMGB1, FDX1, LIAS, DLAT, HIF-1α, VEGF, PD-L1, Caspase-3, ATP7A, β-actin, and the corresponding HRP-conjugated secondary antibodies were purchased from Proteintech Group, Inc. (Wuhan Sanying Biotechnology Co., Ltd., Wuhan, China). Flow cytometry antibodies against CD80, CD86, CD206, CD4, and CD8 were also purchased from Proteintech Group, Inc. (Wuhan, China). All other reagents were of analytical grade and used without further purification.

### 4.2 Synthesis of CuMCDs

CuMCDs were synthesized via a one-pot hydrothermal method. Briefly, 0.5 mmol copper acetate, 1 mmol metformin hydrochloride, and 1 mmol 1,8-naphthalic anhydride were dissolved in 30 mL deionized water, and sonicated for 30 min to form a homogeneous solution. The mixture was transferred to a Teflon-lined autoclave and heated at 180 °C for 6 h. After the reaction, the autoclave was cooled to room temperature naturally. The obtained product was centrifuged at 8000 rpm for 10 min to remove large aggregates, and then dialyzed against deionized water using a dialysis membrane (MWCO 1000 Da) for 3 days to remove unreacted small molecules. Finally, the CuMCDs solution was lyophilized and stored at 4 °C for further use.

### 4.3 Synthesis of HA-CA conjugate

HA-CA was synthesized via amide reaction between the amino group of HA and the aldehyde group of CA. Briefly, 100 mg HA was dissolved in 20 mL deionized water, followed by the addition of 120 mg EDC and 75 mg NHS. The mixture was stirred at room temperature for 1 h to activate the carboxyl groups of HA. Then, 60 mg CA was added to the solution, and the reaction was continued for 24 h under stirring in the dark. The resulting solution was dialyzed against deionized water (MWCO 3500 Da) for 3 days to remove unreacted reagents, and then lyophilized to obtain the HA-CA conjugate. The successful synthesis was verified by FT-IR spectroscopy.

### 4.4 Preparation of CuMCDs@R837 nanoassemblies

CuMCDs@R837 nanoassemblies were prepared via π-π stacking self-assembly. Briefly, 10 mg of CuMCDs (with a copper doping content of 3.43% determined by ICP-MS) and 8 mg of R837 were co-dissolved in 5 mL of DMSO, followed by sonication for 10 min to obtain a homogeneous mixture. The above solution was then added dropwise into 10 mL of deionized water under vigorous stirring. After continuous stirring for 12 h in the dark, the mixture was dialyzed against deionized water (MWCO 3500 Da) for 24 h to remove residual DMSO and unencapsulated free R837. The obtained CuMCDs@R837 suspension was stored at 4 °C for subsequent experiments. The drug loading efficiency of CuMCDs in the nanoassemblies was quantified by detecting the total copper content via inductively coupled plasma mass spectrometry (ICP-MS), while the loading efficiency of R837 was determined by ultraviolet-visible (UV-vis) spectrophotometry based on the standard curve method. The drug loading efficiencies of CuMCDs and R837 were calculated to be 43.6% and 35.2%, respectively.

### 4.5 Preparation of HACCR nanoplatform

HACCR nanoplatform was prepared via the self-assembly of HA-CA and CuMCDs@R837. Briefly, 20 mg HA-CA was dissolved in 10 mL deionized water, and then 5 mL CuMCDs@R837 suspension was added dropwise under sonication. The mixture was sonicated for 30 min and then stirred for 4 h in the dark. The resulting HACCR solution was centrifuged at 3000 rpm for 5 min to remove large aggregates, and then washed with deionized water three times by ultrafiltration (MWCO 100 kDa). The final HACCR suspension was stored at 4 °C for further experiments.

### 4.6 Physicochemical characterization

The morphology of the nanomaterials was characterized by a transmission electron microscope (TEM, JEOL JEM-2100, Japan) at an accelerating voltage of 200 kV. The hydrodynamic size, size distribution, and zeta potential were measured by a dynamic light scattering (DLS) instrument (Malvern Zetasizer Nano ZS90, UK). XRD patterns were recorded on a Bruker D8 Advance X-ray diffractometer (Germany). Raman spectra were obtained using a Renishaw inVia Raman microscope (UK) with a 532 nm laser. FT-IR spectra were recorded on a Nicolet iS50 F T-IR spectrometer (USA) using the KBr pellet method. XPS measurements were performed on a Thermo Scientific ESCALAB 250Xi spectrometer (USA). UV-vis-NIR absorption spectra were recorded on a Shimadzu UV-3600 spectrophotometer (Japan). Fluorescence emission spectra were measured on a Hitachi F-7000 fluorescence spectrophotometer (Japan). ESR spectra were recorded on a Bruker E500 EPR spectrometer (Germany) using 5,5-dimethyl-1-pyrroline N-oxide (DMPO) as the spin trap.

### 4.7 *In vitro* photothermal performance test

The photothermal performance of HACCR was evaluated by irradiating the aqueous solution of HACCR at different concentrations (0, 50, 100, 150, 200μg mL^−1^) with an 808 nm laser (1.0 W cm^−2^) for 10 min. The temperature was recorded every 30 s using a thermocouple, and infrared thermal images were captured by an infrared thermal camera (FLIR, USA). To evaluate the photothermal stability, the HACCR solution (200 μg mL^−1^) was subjected to five laser on-off cycles (10 min laser on, 15 min laser off for cooling). The photothermal conversion efficiency was calculated according to the reported method.

### 4.8 pH-responsive drug release assay

The in vitro release of CuMCDs and R837 from HACCR was investigated using a dialysis method. Briefly, 2 mL of HACCR solution was transferred to a dialysis bag (MWCO 100 kDa), which was immersed in 30 mL of release medium (pH 7.4 PB S or pH 5.5 acetate buffer) and incubated in a shaker at 37 °C with constant shaking. At predetermined time points, 1 mL of the release medium was collected and replaced with an equal volume of fresh medium. The concentration of released CuMCDs was determined by ICP-MS, and the concentration of R837 was measured by UV-vis spectrophotometry. The cumulative release percentage was calculated accordingly.

### 4.9 Colloidal stability assay

HACCR was dispersed in saline, pH 7.4 P BS, and RMPI 1640 medium containing 10% FBS, respectively, and incubated at 37 °C for 7 days. At predetermined time points, the hydrodynamic size, UV absorbance at 808 nm, and zeta potential of the samples were measured to evaluate the colloidal stability.

### 4.10 Cell culture

Mouse colorectal cancer CT26 cells, mouse dendritic DC2.4 cells, and mouse macrophage Raw264.7 cells were purchased from Shanghai Jinyuan Biotechnology Co., Ltd. (Shanghai, China). CT26 and Raw264.7 cells were cultured in RPMI 1640 medium supplemented with 10% fetal bovine serum (FBS), 100 U mL^−1^ penicillin, and 100 μg mL^−1^ streptomycin. DC2.4 cells were cultured in RPMI 1640 medium supplemented with 10% FBS, 1% non-essential amino acids, 1% sodium pyruvate, 50 μM β-mercaptoethanol, 100 U mL^−1^ penicillin, and 100 μg mL^−1^ streptomycin. All cells were incubated in a humidified atmosphere at 37 °C with 5% CO_2_.

### 4.11 Cellular uptake assay

CT26 cells were seeded in 6-well plates containing sterile cell slides at a density of 1 × 10^5^ cells per well and cultured overnight. For the time-dependent uptake experiment, the cells were incubated with HACCR for 0 h, 1 h, 2 h, and 3 h, respectively. For the competitive inhibition experiment, the cells were pre-treated with 10 mg mL^−1^ free HA for 2 h prior to HACCR incubation. After the corresponding incubation, the cells were washed with pre-cooled PBS 3 times, fixed with 4% paraformaldehyde for 15 min, and stained with DAPI for 10 min at room temperature in the dark. Fluorescence images were captured by a fluorescence microscope, and semi-quantitative analysis of the fluorescence intensity was performed using ImageJ software.

### 4.12 Mitochondria Co-localization assay

CT26 cells were seeded in 6-well plates containing sterile cell slides at a density of 1 × 10^5^ cells per well and cultured overnight, followed by incubation with HACCR for 3 h. Subsequently, the cells were washed with pre-cooled PBS, and stained with MitoTracker Green (200 nM) for 30 min at 37 °C in the dark. After washing with pre-cooled PBS, the cells were fixed with 4% paraformaldehyde and stained with DAPI as described above. Fluorescence images were acquired using a fluorescence microscope, the co-localization between HACCR and mitochondria was analyzed, and the Pearson correlation coefficient was calculated via ImageJ software.

### 4.13 Cell viability assay

The cytotoxicity of HACCR was evaluated by the CCK-8 assay. CT26 cells were seeded in 96-well plates at a density of 5 × 10^3^ cells per well and cultured overnight. The cells were treated with different concentrations of HACCR (0, 50, 100, 150, 200, 300 μg mL^−1^) for 24 h. For the photothermal group, the cells were irradiated with an 808 nm laser (1.0 W cm^−2^, 5 min) after 4 h of incubation with HACCR. Then, 10 μL of CCK-8 solution was added to each well, and the plates were incubated for another 2 h. The absorbance at 450 nm was measured using a microplate reader (Tecan, Switzerland). The relative cell viability was calculated as (ODtreatment/ODcontrol) × 100%. The cytotoxicity of free CuSO_4_ and the reversal effect of TTM were also evaluated using the same method.

### 4.14 Live/dead cell staining

CT26 cells were seeded in 6-well plates containing sterile cell slides at a density of 1 × 10^5^ cells per well and cultured overnight, then treated with gradient concentrations of HACCR with or without laser irradiation. After 24 h of incubation, the cells were washed with pre-cooled PBS 3 times, and stained with Calcein-AM and PI for 15 min at 37 °C in the dark. Fluorescence images were then captured using a fluorescence microscope. Cells treated with CuSO_4_ were prepared in parallel as the control group.

### 4.15 Intracellular ROS detection

CT26 cells were seeded in 6-well plates containing sterile cell slides at a density of 1 × 10^5^ cells per well and cultured overnight, then treated with gradient concentrations of HACCR with or without laser irradiation. After the corresponding incubation, the cells were washed with pre-cooled PBS 3 times, and incubated with DCFH-DA (10 μM) for 20 min at 37 °C in the dark. Subsequently, the cells were washed thoroughly with pre-cooled PBS, and fluorescence images were captured using a fluorescence microscope. Semi-quantitative analysis of the fluorescence intensity was performed using ImageJ software.

### 4.16 Intracellular Cu (I) detection

The intracellular Cu (I) level was detected using a Cu (I)-specific fluorescent probe. CT26 cells were treated with different concentrations of HACCR for 12 h, then washed with PBS and incubated with the Cu (I) probe for 30 min. The cells were observed under CLSM, and the fluorescence intensity was semi-quantified by ImageJ.

### 4.17 Immunofluorescence staining

CT26 cells were seeded in 6-well plates containing sterile cell slides at a density of 1 × 10^5^ cells per well and cultured overnight, then treated with gradient concentrations of HACCR for 24 h. For the TTM reversal experiment, cells were pre-treated with 10 μM TTM for 2 h prior to HACCR administration. After the corresponding treatment, cells were washed with pre-cooled PBS 3 times, fixed with 4% paraformaldehyde for 15 min, permeabilized with 0.1% Triton X-100 for 15 min at room temperature, and blocked with 5% BSA for 1 h at room temperature. Subsequently, cells were incubated with primary antibodies against FDX1, LIAS, or DLAT overnight at 4 °C, followed by incubation with fluorescently labeled secondary antibodies for 1 h at room temperature in the dark. Cells were washed with PBS 3 times between each incubation step. After nuclear counterstaining with DAPI in the dark, fluorescence images were acquired using a fluorescence microscope, and semi-quantitative analysis of the target fluorescence intensity was performed via ImageJ software.

### 4.18 Mitochondrial membrane potential detection

The mitochondrial membrane potential was detected using the JC-1 probe. CT26 cells were treated with different concentrations of HACCR for 12 h, then washed with PBS and incubated with JC-1 working solution for 20 min at 37 °C. The cells were washed and observed under CLSM. The red and green fluorescence intensity was semi-quantified using ImageJ.

### 4.19 WB analysis

Cells or tumor tissues were lysed with RIPA lysis buffer containing protease and phosphatase inhibitors. The total protein concentration was determined using the BCA kit. Equal amounts of protein samples were separated by SDS-PAGE and transferred to PVDF membranes. The membranes were blocked with 5% skim milk for 1 h, and then incubated with primary antibodies overnight at 4 °C. After washing with TBST, the membranes were incubated with HRP-conjugated secondary antibodies for 1 h at room temperature. The protein bands were visualized using an enhanced chemiluminescence (ECL) kit, and the gray value was quantified using ImageJ software.

### 4.20 Intracellular metabolite detection

The intracellular levels of GSH, pyruvate, and α-KG were measured using corresponding commercial assay kits (Beyotime, China) according to the manufacturer's instructions. CT26 cells were treated with different concentrations of HACCR for 24 h, then lysed and centrifuged to collect the supernatant. The absorbance was measured using a microplate reader, and the metabolite content was calculated according to the standard curve.

### 4.21 *In vitro* ICD and immune cell Co-incubation assay

CT26 cells were treated with Saline, HACCR, or HACCR + Laser for 24 h. The culture supernatant was collected as the conditioned medium, and the treated cells were collected for WB analysis of ICD-related proteins. The extracellular ATP level in the supernatant was measured using a commercial assay kit.

For DC maturation assay, immature DC2.4 cells were co-incubated with the differently treated CT26 cells for 24 h. Then, the DCs were collected, stained with fluorescently labeled anti-CD11c, anti-CD80, and anti-CD86 antibodies, and analyzed by flow cytometry.

For macrophage polarization assay, Raw264.7 cells were co-incubated with the conditioned medium of differently treated CT26 cells for 24 h. Then, the macrophages were collected, stained with anti-F4/80, anti-CD86, and anti-CD206 antibodies, and analyzed by flow cytometry.

### 4.22 Cytokine secretion assay

DC2.4 cells or Raw264.7 cells were treated as described above. The culture supernatant was collected, and the levels of IL-6, TNF-α, and IL-10 were measured using enzyme-linked immunosorbent assay (ELISA) kits (R&D Systems, USA) according to the manufacturer's instructions.

### 4.23 Animal experiments

All animal experiments in this study were approved by the Animal EthicsCommittee of Harbin Normal University (approval No. HNUARIA2023003). All procedures were performed in strict compliance with international and institutional guidelines for the proper care and humane use of laboratory animals. Hou-sing, handling, and disposal of animals fully met ethical and safety standards. This study involved no human participants or human biological samples, only

Laboratory animals and cell lines. No unexpected or unusually high safety hazards occurred during the entire study. All experiments were carried out under standard laboratory safety protocols. Reagents, instruments, and workflows underwent routine safety evaluation and control, with no novel or major risks identi-fied.

Female Balb/c mice (6-8 weeks old) were purchased from the Laboratory Animal Center of Harbin Medical University. For the *in vivo* imaging experiment, CT26 tumor-bearing mice were established by subcutaneously inoculating 1 × 10^6^ CT26 cells into the right flank of the mice. When the tumor volume reached ∼100 mm^3^, the mice were intravenously injected with HACCR. At predetermined time points (0, 2, 4, 8, 12, 24, 48 h post-injection), the *in vivo* fluorescence images were captured using a small animal *in vivo* imaging system (IVIS Lumina III, PerkinElmer, USA). For *in vivo* photothermal imaging, the tumor region of the mice was irradiated with an 808 nm laser (1.0 W cm^−2^) for 10 min at different time points post-injection, and the infrared thermal images were recorded by an infrared thermal camera. At 48 h post-injection, the mice were sacrificed, and the major organs (heart, liver, spleen, lung, kidney) and tumor tissues were collected for ex vivo fluorescence imaging.

For the *in vivo* antitumor experiment, the bilateral CT26 tumor-bearing mouse model was established by subcutaneously inoculating 5 × 10^5^ CT26 cells into the right flank (primary tumor) and 2 × 10^5^ CT26 cells into the left flank (distant tumor) of each mouse. When the primary tumor volume reached ∼100 mm^3^, the mice were randomly divided into 5 groups (n = 5 per group): (1) Saline, (2) αPD-L1 (100 μg per mouse), (3) HACCR (10 mg kg^−1^ CuMCDs equivalent), (4) HACCR + Laser, (5) HACCR + Laser + αPD-L1. The drugs were administered via tail vein injection on day 0, 5, and 10. For the laser irradiation groups, the primary tumor was irradiated with an 808 nm laser (1.0 W cm^−2^) for 10 min at 8 h post-injection. αPD-L1 was administered via intraperitoneal injection on day 1, 6, and 11. The tumor volume and body weight of the mice were recorded every 2 days. The tumor volume was calculated as (length × width^2^)/2. At the end of the treatment (day 14), the mice were sacrificed, and the primary and distant tumors were collected, photographed, and weighed.

### 4.24 *In vivo* biosafety evaluation

At the end of the treatment, the blood of the mice was collected for blood biochemistry and hematology tests. The major organs (brain, heart, liver, spleen, lung, kidney) and tumor tissues were collected, fixed with 4% paraformaldehyde, embedded in paraffin, sectioned, and stained with H&E for histopathological analysis.

### 4.25 *In vivo* immune mechanism analysis

At the end of the treatment, the TDLNs and tumor tissues were collected, and the single-cell suspension was prepared. The cells were stained with corresponding fluorescent antibodies for DC maturation, macrophage polarization, and T cell subsets analysis by flow cytometry. The tumor tissues were also lysed for WB analysis of immune-related proteins, and the levels of cytokines (IL-6, TNF-α, IFN-γ, IL-10) in the tumor homogenate were measured by ELISA.

## 5 Statistical analysis

All data were presented as mean ± standard deviation (SD). Statistical analysis was performed using GraphPad Prism 8.0 software. The statistical significance between two groups was analyzed by Student's t-test, and multiple groups were compared by one-way analysis of variance (ANOVA). *P* < 0.05 was considered statistically significant (∗*P* < 0.05, ∗∗*P* < 0.01, ∗∗∗*P* < 0.001; ns: not significant).

## CRediT authorship contribution statement

**Hui Zhang:** Data curation, Formal analysis, Investigation, Methodology, Writing – original draft. **Yuting Lu:** Data curation, Formal analysis, Investigation, Methodology, Writing – original draft. **Jingchun Wang:** Data curation, Formal analysis. **Yikai Ma:** Data curation, Formal analysis. **Yuanjin Sun:** Data curation, Formal analysis. **Shengzhong Rong:** Funding acquisition, Project administration. **Xu Zhu:** Conceptualization, Funding acquisition, Methodology, Writing – review & editing. **Yingxue Jin:** Funding acquisition, Project administration.

## Declaration of competing interest

The authors declare that they have no known competing financial interests or personal relationships that could have appeared to influence the work reported in this paper.

## Data Availability

Data will be made available on request.

## References

[1] Li X., Shen M., Yang J., Liu L., Yang Y.W. (2024). Pillararene-based stimuli-responsive supramolecular delivery systems for cancer therapy. Adv. Mater..

[2] Zheng X., Zhuang J., Peng X., Zhang Y., Chen D., Chen B. (2025). DNA nanotherapeutics facilitate efficient gynecological cancers treatment. J. Contr. Release.

[3] Sun Y., Lian T., Huang Q., Chang Y., Li Y., Guo X., Kong W., Yang Y., Zhang K., Wang P., Wang X. (2023). Nanomedicine-mediated regulated cell death in cancer immunotherapy. J. Contr. Release.

[4] Meng Y., Chen Q., Zhou Z., Li M. (2025). Regulated cell death in cancer: mechanisms, crosstalk, and opportunities for therapy. Cancer Lett..

[5] Tsvetkov P., Coy S., Petrova B., Dreishpoon M., Verma A., Abdusamad M., Rossen J., Joesch-Cohen L., Humeidi R., Spangler R.D., Eaton J.K., Frenkel E., Kocak M., Corsello S.M., Lutsenko S., Kanarek N., Santagata S., Golub T.R. (2022). Copper induces cell death by targeting lipoylated TCA cycle proteins. Science.

[6] Li E., Wen L., Yin C., Wang N., Yang S., Feng W., Chen M. (2025). Copper ionophore-autophagy interference nanoregulators for tumor self-defense reprograming to amplify cuproptotic stress and antitumor immunity. J. Contr. Release.

[7] Hao Q., Gan Y., Zhou X. (2026). Tackling cuproptosis: from metabolic rewiring to therapeutic exploitation in cancer. Cell. Mol. Immunol..

[8] Chen W., Lin C., Gao Z., Huang Y., Wang X., Zhang Q., Zhang Y., Tan M., Hou Z. (2025). A tumor microenvironment-responsive nanocomposite for enhanced copper retention and hypoxia reversal to promote cuproptosis in tumor treatment. Acta Biomater..

[9] Deng J., Zhuang H., Shao S., Zeng X., Xue P., Bai T., Wang X., Shangguan S., Chen Y., Yan S., Huang W. (2024). Mitochondrial-targeted copper delivery for cuproptosis-based synergistic cancer therapy. Adv. Healthcare Mater..

[10] Tang C., Liu K., Gao X., Kang H., Xie W., Chang J., Yin L., Kang J. (2025). A metal-organic framework functionalized CaO_2_-based Cascade nanoreactor induces synergistic cuproptosis/ferroptosis and Ca^2+^ overload-mediated mitochondrial damage for enhanced sono-chemodynamic immunotherapy. Acta Biomater..

[11] Bai J., Zhang X., Zhao Z., Sun S., Cheng W., Yu H., Chang X., Wang B. (2024). CuO nanozymes catalyze cysteine and glutathione depletion induced ferroptosis and cuproptosis for synergistic tumor therapy. Small.

[12] Xiong Y., Yong Z., Xu C., Deng Q., Wang Q., Li S., Wang C., Zhang Z., Yang X., Li Z. (2023). Hyperbaric oxygen activates enzyme-driven Cascade reactions for cooperative cancer therapy and cancer stem cells elimination. Adv. Sci..

[13] Jiang T., Jia T., Yin Y., Li T., Song X., Feng W., Wang S., Ding L., Chen Y., Zhang Q. (2025). Cuproptosis-inducing functional nanocomposites for enhanced and synergistic cancer radiotherapy. ACS Nano.

[14] Liu C., Guo L., Cheng Y., Gao J., Pan H., Zhu J., Li D., Jiao L., Fu C. (2025). A mitochondria-targeted nanozyme platform for multi-pathway tumor therapy via ferroptosis and cuproptosis regulation. Adv. Sci..

[15] Zhao F., Yu H., Liang L., Wang C., Shi D., Zhang X., Ying Y., Cai W., Li W., Li J., Zheng J., Qiao L., Che S., Yu J. (2023). Redox homeostasis disruptors based on metal-phenolic network nanoparticles for chemo/chemodynamic synergistic tumor therapy through activating apoptosis and cuproptosis. Adv. Healthcare Mater..

[16] Chen W., Xie W., Gao Z., Lin C., Tan M., Zhang Y., Hou Z. (2023). Mild-photothermal effect induced high efficiency ferroptosis-boosted-cuproptosis based on Cu_2_O@Mn_3_Cu_3_O_8_ nanozyme. Adv. Sci..

[17] Mei J., Pan W., Li B., Zhong M., Shi X., Cheng Y., Wu B., Xiu Q., Xue Y., Situ B., Zheng L. (2025). Photosynthetic plant-derived nanovesicles precisely amplify photodynamic effect by light-activated oxygen generation for enhanced cancer photoimmunotherapy. ACS Nano.

[18] Liang J.L., Jin X.K., Luo G.F., Zhang S.M., Huang Q.X., Lin Y.T., Deng X.C., Wang J.W., Chen W.H., Zhang X.Z. (2023). Immunostimulant hydrogel-guided tumor microenvironment reprogramming to efficiently potentiate macrophage-mediated cellular phagocytosis for systemic cancer immunotherapy. ACS Nano.

[19] Huang X., Mu N., Ding Y., Lam H.W., Yue L., Gao C., Chen T., Yuan Z., Wang R. (2022). Targeted delivery and enhanced uptake of chemo-photodynamic nanomedicine for melanoma treatment. Acta Biomater..

[20] Elzoghby A.O., Hemasa A.L., Freag M.S. (2016). Hybrid protein-inorganic nanoparticles: from tumor-targeted drug delivery to cancer imaging. J. Contr. Release.

[21] Ghirardello M., Ramos-Soriano J., Galan M.C. (2025). Carbon dots as an emergent class of sustainable antifungal agents. ACS Nano.

[22] Alafeef M., Srivastava I., Aditya T., Pan D. (2024). Carbon dots: from synthesis to unraveling the fluorescence mechanism. Small.

[23] Li J., Gong X. (2022). The emerging development of multicolor carbon dots. Small.

[24] Wang X., Li X., Mao Y., Wang D., Zhao Q., Wang S. (2019). Multi-stimuli responsive nanosystem modified by tumor-targeted carbon dots for chemophototherapy synergistic therapy. J. Colloid Interface Sci..

[25] Zhu X., Lu Y., Sun M., Wang Z., Jin S., Ma M., Wu X., Zhang H., Geng F., Jin Y. (2026). TLR agonist/copper-based metformin carbon dot-loaded homologous membrane nanocarriers with enhanced immunogenic cell death for synchronous amelioration of tumor microenvironment hypoxia, immunosuppression, and metastasis inhibition. ACS Appl. Mater. Interfaces.

[26] Lahooti B., Akwii R.G., Zahra F.T., Sajib M.S., Lamprou M., Alobaida A., Lionakis M.S., Mattheolabakis G., Mikelis C.M. (2023). Targeting endothelial permeability in the EPR effect. J. Contr. Release.

[27] Guo Z., Liang E., Sui J., Ma M., Yang L., Wang J., Hu J., Sun Y., Fan Y. (2020). Lapatinib-loaded acidity-triggered charge switchable polycarbonate-doxorubicin conjugate micelles for synergistic breast cancer chemotherapy. Acta Biomater..

[28] He C., Zhang Y., Liu X., Yu C., Lv J., Lin Y., Zhao L., Li M. (2026). Self-amplifying hypoxia Cascade in covalent organic framework/metal organic framework nanoreactors for synergistic cancer therapy. J. Colloid Interface Sci..

[29] He M., Zhang M., Xu T., Xue S., Li D., Zhao Y., Zhi F., Ding D. (2024). Enhancing photodynamic immunotherapy by reprograming the immunosuppressive tumor microenvironment with hypoxia relief. J. Contr. Release.

[30] Tao N., Li H., Deng L., Zhao S., Ouyang J., Wen M., Chen W., Zeng K., Wei C., Liu Y.N. (2022). A Cascade nanozyme with amplified sonodynamic therapeutic effects through comodulation of hypoxia and immunosuppression against cancer. ACS Nano.

[31] Zeng S.M., Qu W.Q., Sun Y.L., Chen K.W., Zhao K., Yan J.H., Zhang C., Liang C.X., Chen Y., Pan T., Yu A., Zhang X.Z. (2025). MnO_2_-assisted photosynthetic bacteria interfering with the adenosine-A2AR metabolic pathway to enhance tumor photothermal immunotherapy. ACS Nano.

[32] Chen Y., Wang J., Zheng Y., Zhang J., Wang J., Ye H., Tao L., Yao Y., Luo X., Ding Y., Shen X. (2025). Oligocopper-loaded lipoic acid nanoparticles promote mitochondrial protein lipoylation for augmented cuproptosis therapy. Small.

[33] Lu X., Deng W., Wang S., Zhao S., Zhu B., Bai B., Mao Y., Lin J., Yi Y., Xie Z., Wang X., Lu Y., Huang X., You T., Chen X., Sun W., Shen X. (2024). PEGylated Elesclomol@Cu(Ⅱ)-based metal‒organic framework with effective nanozyme performance and cuproptosis induction efficacy for enhanced PD-L1-based immunotherapy. Mater. Today Bio.

[34] Wang N., Liu Y., Peng D., Zhang Q., Zhang Z., Xu L., Yin L., Zhao X., Lu Z., Peng J. (2024). Copper-based composites nanoparticles improve triple-negative breast cancer treatment with induction of apoptosis-cuproptosis and immune activation. Adv. Healthcare Mater..

[35] Zeng Y., Cao Y., Ren S., Zhang C., Liu J., Liu K., Wang Y., Chen H., Zhou F., Yang X., Ge X., Zhang T., Wang T., He Y., Li D., Zhang C., Lu J. (2025). Responsive ROS-Augmented prodrug hybridization nanoassemblies for multidimensionally synergitic treatment of hepatocellular carcinoma in Cascade assaults. Adv. Sci..

[36] Li J., Ma S., Lin Q., Wang Q., Zhong W., Wei C., Liu J., Chen J., Wang D., Tang W., Luo T. (2024). Orchestrated copper-loaded nanoreactor for simultaneous induction of cuproptosis and immunotherapeutic intervention in colorectal cancer. Mater. Today Bio.

[37] Du H., Li Z., Fu S.T., Yang C.J., Yin Y.Z., Chen Z., Zhao C.Q., Qiao H., Ji D.K. (2025). Mitochondria-targeted dual-ion perturbator amplifies cuproptosis for enhanced melanoma immunotherapy and accelerated postoperative wound healing. ACS Nano.

[38] Yang Y., Zhu Y., Wang K., Miao Y., Zhang Y., Gao J., Qin H., Zhang Y. (2023). Activation of autophagy by in situ Zn^2+^ chelation reaction for enhanced tumor chemoimmunotherapy. Bioact. Mater..

[39] Deng T., Chen D., Chen F., Xu C., Zhang Q., Li M., Wang Y., He Z., Li M., He Q. (2024). Synergizing autophagic cell death and oxaliplatin-induced immunogenic death by a self-delivery micelle for enhanced tumor immunotherapy. Acta Biomater..

[40] Li Q., Chen C., Kong J., Li L., Li J., Huang Y. (2022). Stimuli-responsive nano vehicle enhances cancer immunotherapy by coordinating mitochondria-targeted immunogenic cell death and PD-L1 blockade. Acta Pharm. Sin. B.

[41] Lin M., Cai Y., Chen G., Zhong H., Li B., Li T., Xiao Z., Shuai X. (2023). A hierarchical tumor-targeting strategy for eliciting potent antitumor immunity against triple negative breast cancer. Biomaterials.

[42] Ghislat G., Lawrence T. (2018). Autophagy in dendritic cells, cell. Mol. Immunol..

[43] Liu H., Lv Z., Zhang G., Yan Z., Bai S., Dong D., Wang K. (2024). Molecular understanding and clinical aspects of tumor-associated macrophages in the immunotherapy of renal cell carcinoma. J. Exp. Clin. Cancer Res..

