# Supplementary material for: Mitochondria-targeted cuproptosis-driven nanoplatform for synergistic photothermal/chemodynamic therapy and systemic antitumor immunotherapy
Source: Mater Today Bio. 2026 May 30;38:103303. doi: 10.1016/j.mtbio.2026.103303 (PMC13253154; doi:10.1016/j.mtbio.2026.103303)
Supplement: Multimedia component 1 [file mmc1.docx]

**Supporting Information**

**Mitochondria-Targeted Cuproptosis-Driven Nanoplatform for Synergistic Photothermal/Chemodynamic Therapy and Systemic Antitumor Immunotherapy**

Hui Zhang ^a^ Yuting Lu ^b^ Jingchun Wang ^c^ Yikai Ma ^b^ Yuanjin Sun ^b^  Shengzhong Rong ^a^

Xu Zhu ^b^ Yingxue Jin ^b^

*^a^ College of Public Health, Mudanjiang Medical University, Mudanjiang, 157009, China*

*^b^ Key Laboratory for Photonic and Electronic Bandgap Materials, Ministry of Education, College of Chemistry & Chemical Engineering, Harbin Normal University, Harbin, 150025, China.*

*^c^ Heilongjiang Nursing College, Harbin, 150025, China.*

*Corresponding authors：*

*Shengzhong Rong: rongsz2026@163.com*

*Xu Zhu: 18715529155@163.com*

*Yingxue Jin: jyxprof@163.com*

**Table of Contents**

**1 Figure S1. EDS elemental mapping**

**2 Figure S2. Characterization of pH-responsive degradation and drug release behavior of HACCR nanoplatform**

**3 Figure S3. In vitro colloidal stability characterization of HACCR nanoparticles**

**4 Figure S4. Relative viability of CT26 cells treated with gradient concentrations of CuSO₄**

**5 Figure S5. Confocal laser scanning microscopy images of live/dead staining of CT26 cells**

**6 Figure S6. Immunofluorescence staining images of FDX1**

**7 Figure S7. Immunofluorescence staining images of LIAS**

**8 Figure S8. Immunofluorescence staining images of cuproptosis-related protein DLAT in CT26 cells**

**9 Figure S9. JC-1 staining images of CT26 cells with different treatments**

**10 Figure S10. In vivo tumor targeting distribution and photothermal performance evaluation of HACCR in tumor-bearing mice**

**11 Figure S11. Hematoxylin and eosin (H&E) staining images of major organs**

**12 Figure S12. Hematoxylin and eosin (H&E) staining images of Brain**

**13 Figure S13. Blood biochemistry and hematology test results of mice in different treatment groups**

**14 Table S1. ICP-MS Detection of Copper Ion Content in Feces**

**1 Figure S1 Figure S1. EDS elemental mapping**


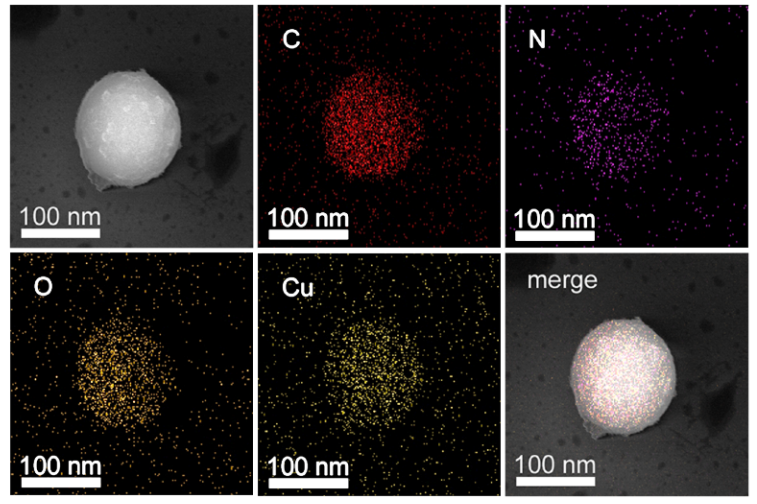


Figure S1. Corresponding EDS elemental mapping (C, N, O, Cu) of HACCR nanoparticles, verifying the homogeneous elemental distribution within the nanoparticles.

**2 Figure S2. Characterization of pH-responsive degradation and drug release behavior of HACCR nanoplatform**


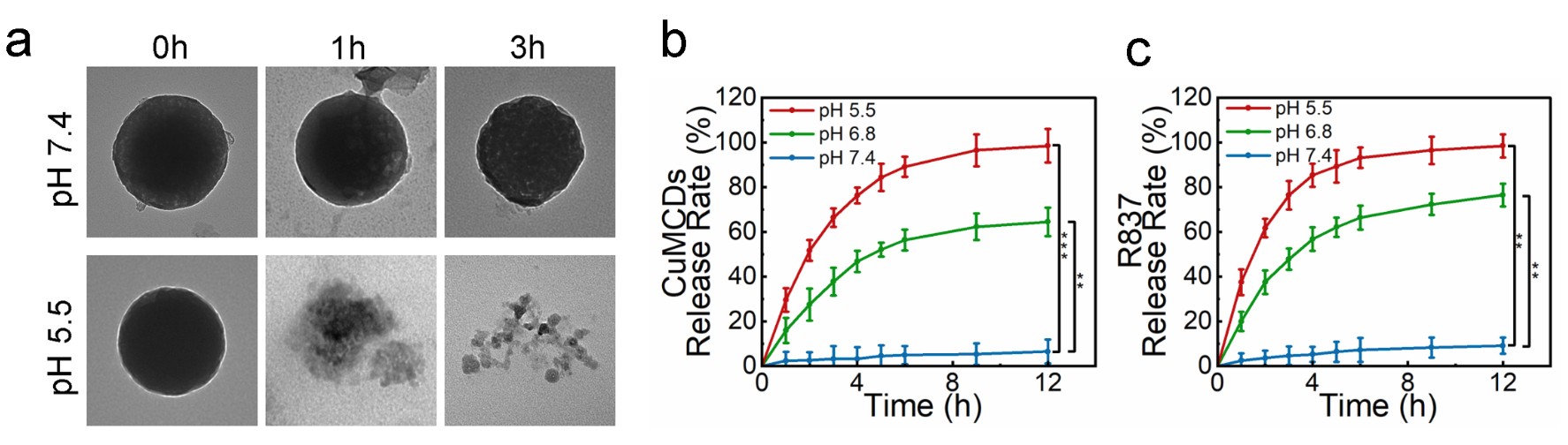


Figure S2. Characterization of pH-responsive degradation and drug release behavior of HACCR nanoplatform. (a) TEM images of HACCR after incubation for 0 h, 1 h, and 3 h at pH 7.4 (physiological condition) and pH 5.5 (acidic tumor microenvironment). (b) Cumulative release profiles of CuMCDs under different pH conditions. (c) Cumulative release profiles of R837 under different pH conditions. Data are presented as mean ± SD (n = 3). Statistical differences were determined by one-way ANOVA.

**3 Figure S3. *In vitro* colloidal stability characterization of HACCR nanoparticles**


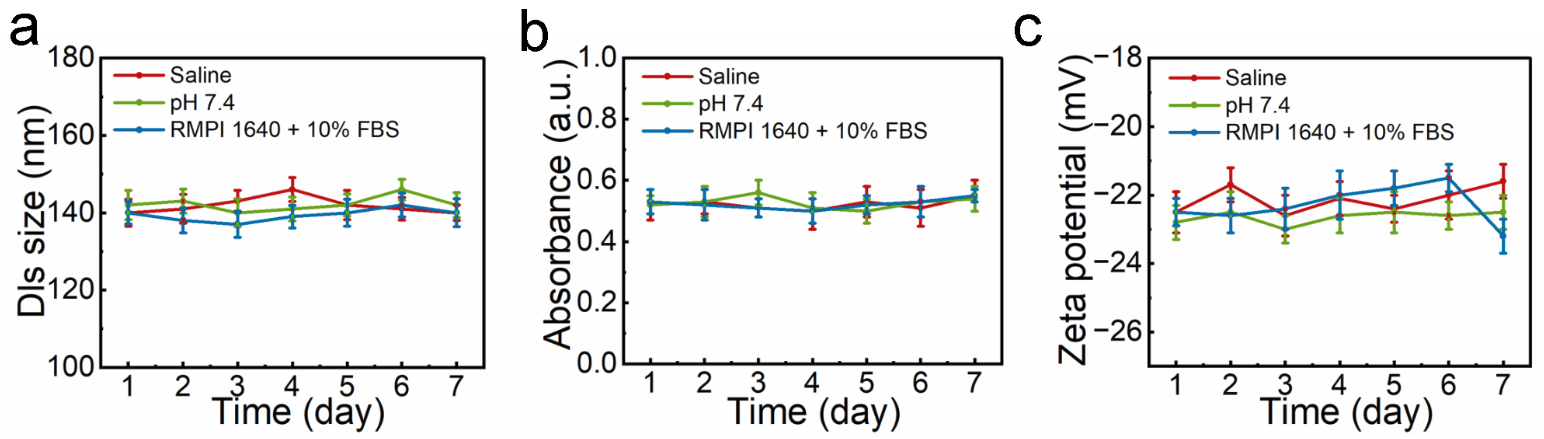


Figure S3. *In vitro* colloidal stability characterization of HACCR nanoparticles. (a) Time-dependent hydrodynamic size changes of HACCR in saline, pH 7.4 buffer, and RMPI 1640 medium with 10% fetal bovine serum (FBS) within 7 days. (b) Time-dependent UV absorbance changes of HACCR in the corresponding media. (c) Time-dependent zeta potential changes of HACCR in the corresponding media. Data are presented as mean ± SD (n = 3). Statistical differences were determined by one-way ANOVA.

**4 Figure S4. Relative viability of CT26 cells treated with gradient**


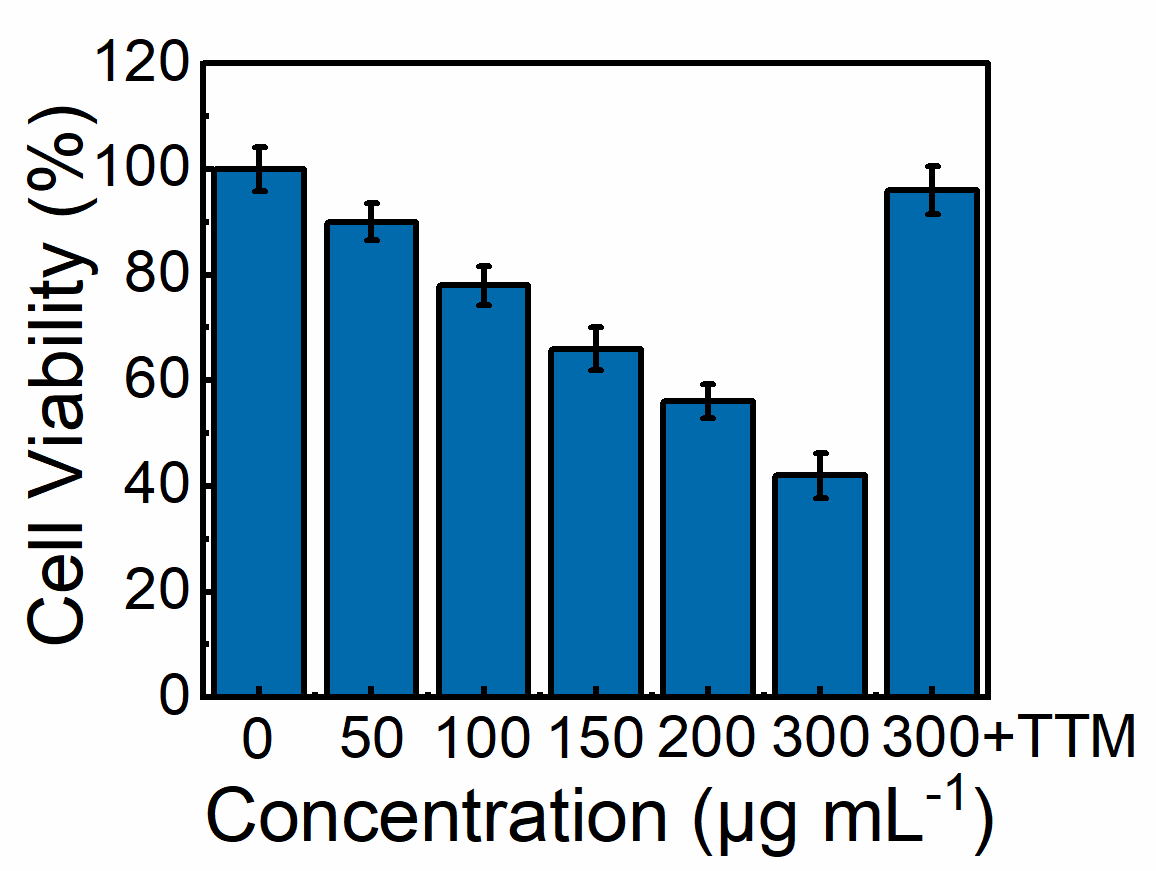


Figure S4. Relative viability of CT26 cells treated with gradient concentrations of CuSO₄, and the reversal effect on cytotoxicity by pretreatment with copper ion chelator TTM. Data are presented as mean ± SD (n = 3). Statistical differences were determined by one-way ANOVA.

**5 Figure S5. Confocal laser scanning microscopy images of live/dead staining of CT26 cells**


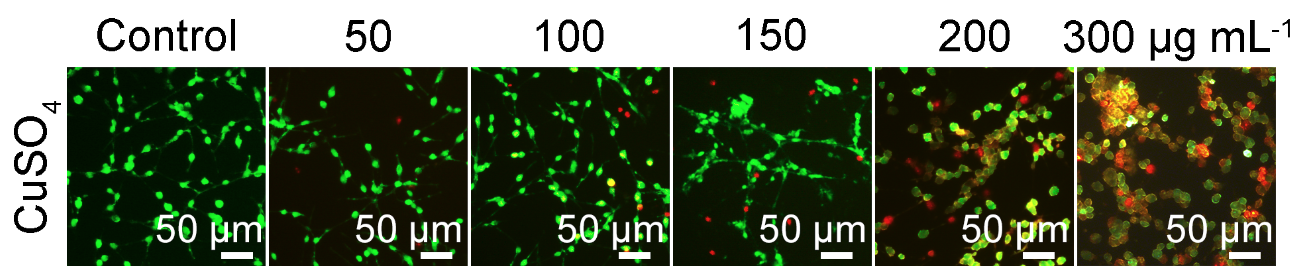


Figure S5. Confocal laser scanning microscopy images of live/dead staining of CT26 cells treated with gradient concentrations of CuSO₄. Live cells are shown with green fluorescence, and dead cells are shown with red fluorescence. Fluorescence quantification was performed using Image J.

**6 Figure S6. Immunofluorescence staining images of FDX1**


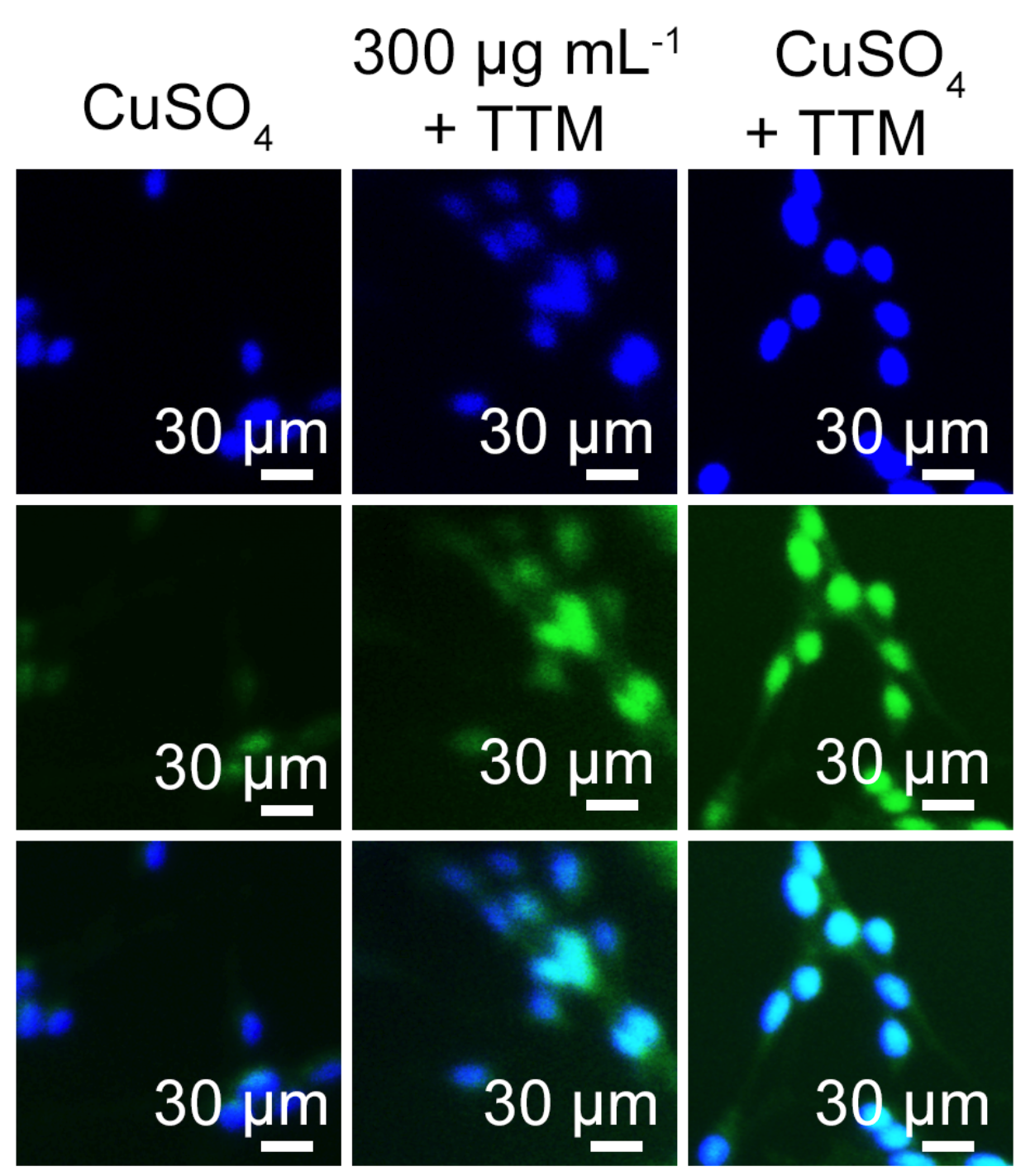


Figure S6. Immunofluorescence staining images of FDX1 (key cuproptosis-related protein) in CT26 cells with different treatments. Blue: DAPI staining for cell nuclei; green: fluorescence signal of FDX1 protein. Fluorescence quantification was performed using Image J.

**7 Figure S7. Immunofluorescence staining images of LIAS**


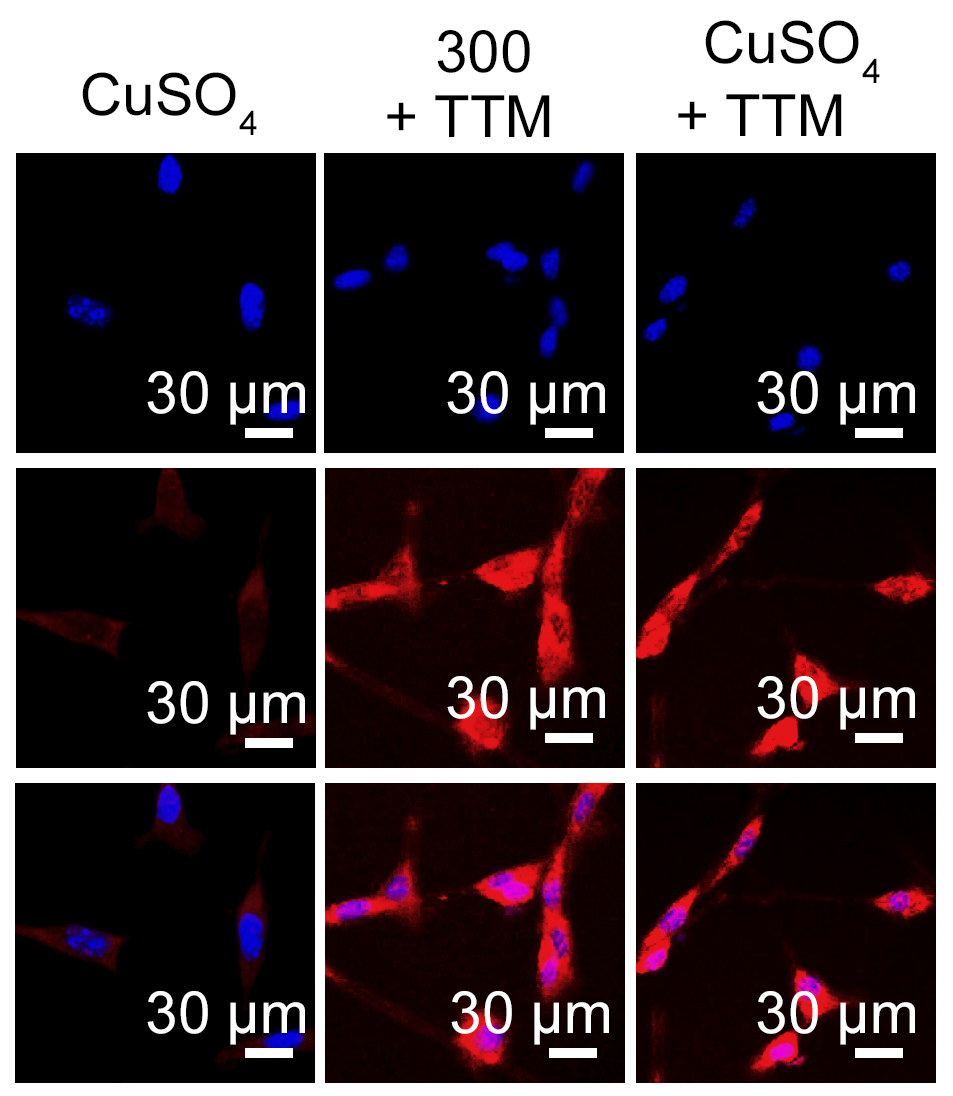


Figure S7. Immunofluorescence staining images of LIAS (key cuproptosis-related protein) in CT26 cells with different treatments. Blue: DAPI staining for cell nuclei; red: fluorescence signal of LIAS protein. Fluorescence quantification was performed using Image J.

**8 Figure S8. Immunofluorescence staining images of cuproptosis-**

**related protein DLAT in CT26 cells**


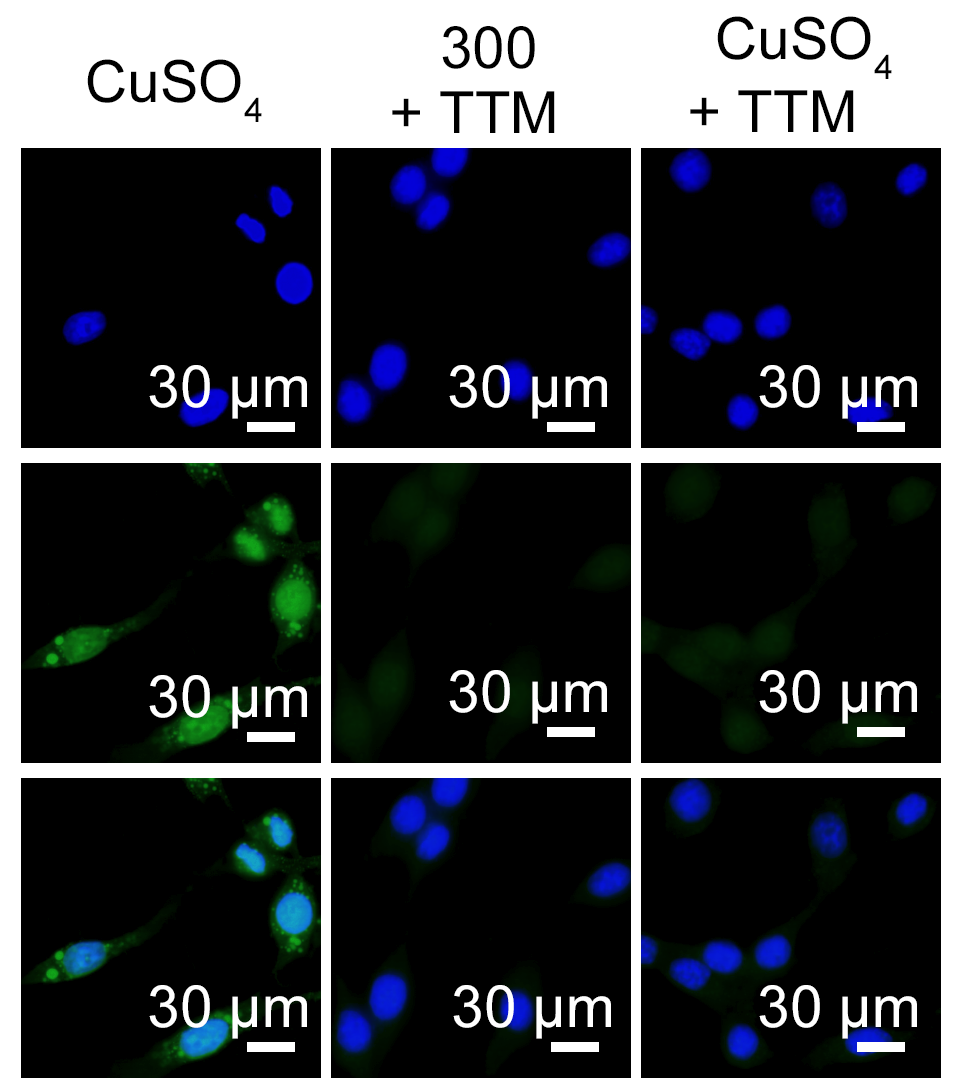


Figure S8. Immunofluorescence staining images of cuproptosis-related protein DLAT in CT26 cells with different treatments. Blue: DAPI staining for cell nuclei; green: fluorescence signal of DLAT protein. Fluorescence quantification was performed using Image J.

**9 Figure S9. JC-1 staining images of CT26 cells with different treatments**


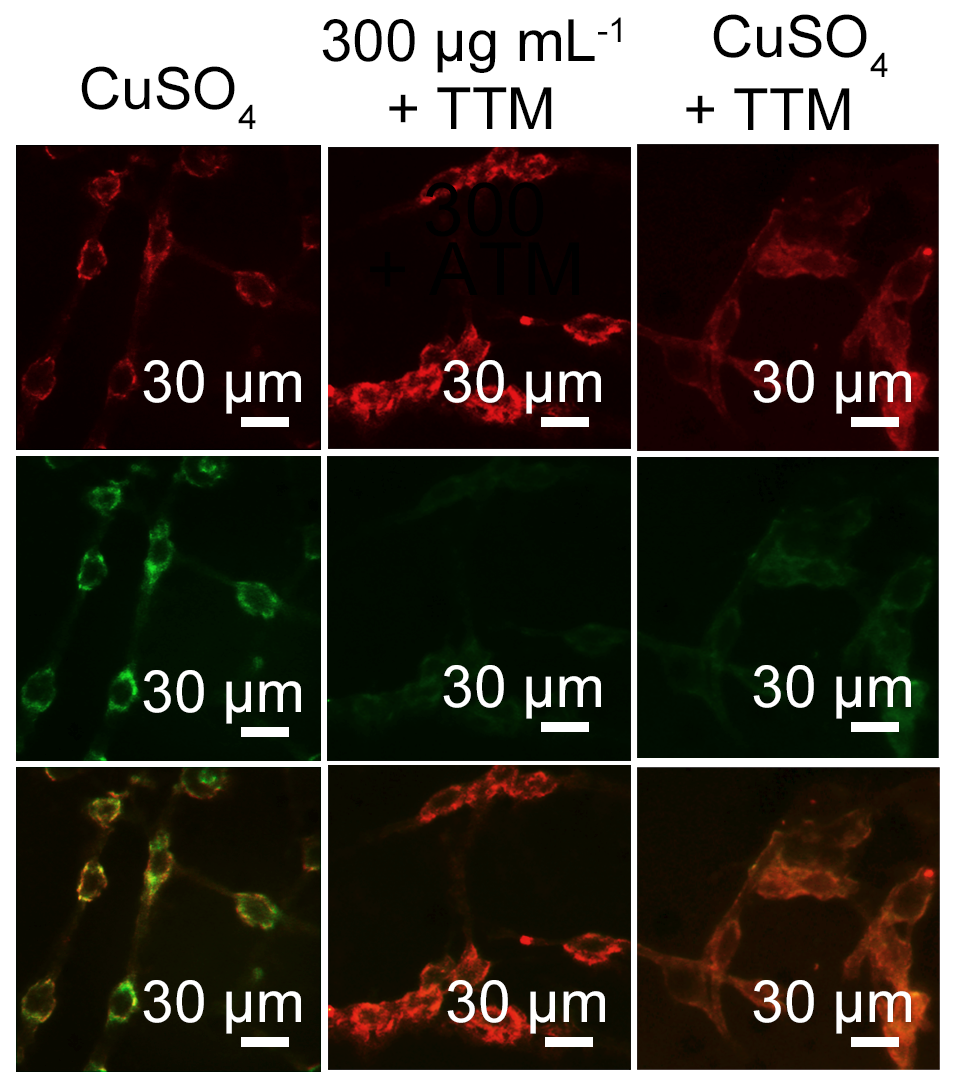


Figure S9. JC-1 staining images of CT26 cells with different treatments. Red fluorescence: JC-1 aggregates; green fluorescence: JC-1 monomers, for detecting mitochondrial membrane potential changes. Fluorescence quantification was performed using Image J.

**10 Figure S10. *In vivo* tumor targeting distribution and photothermal performance evaluation of HACCR in tumor-bearing mice**


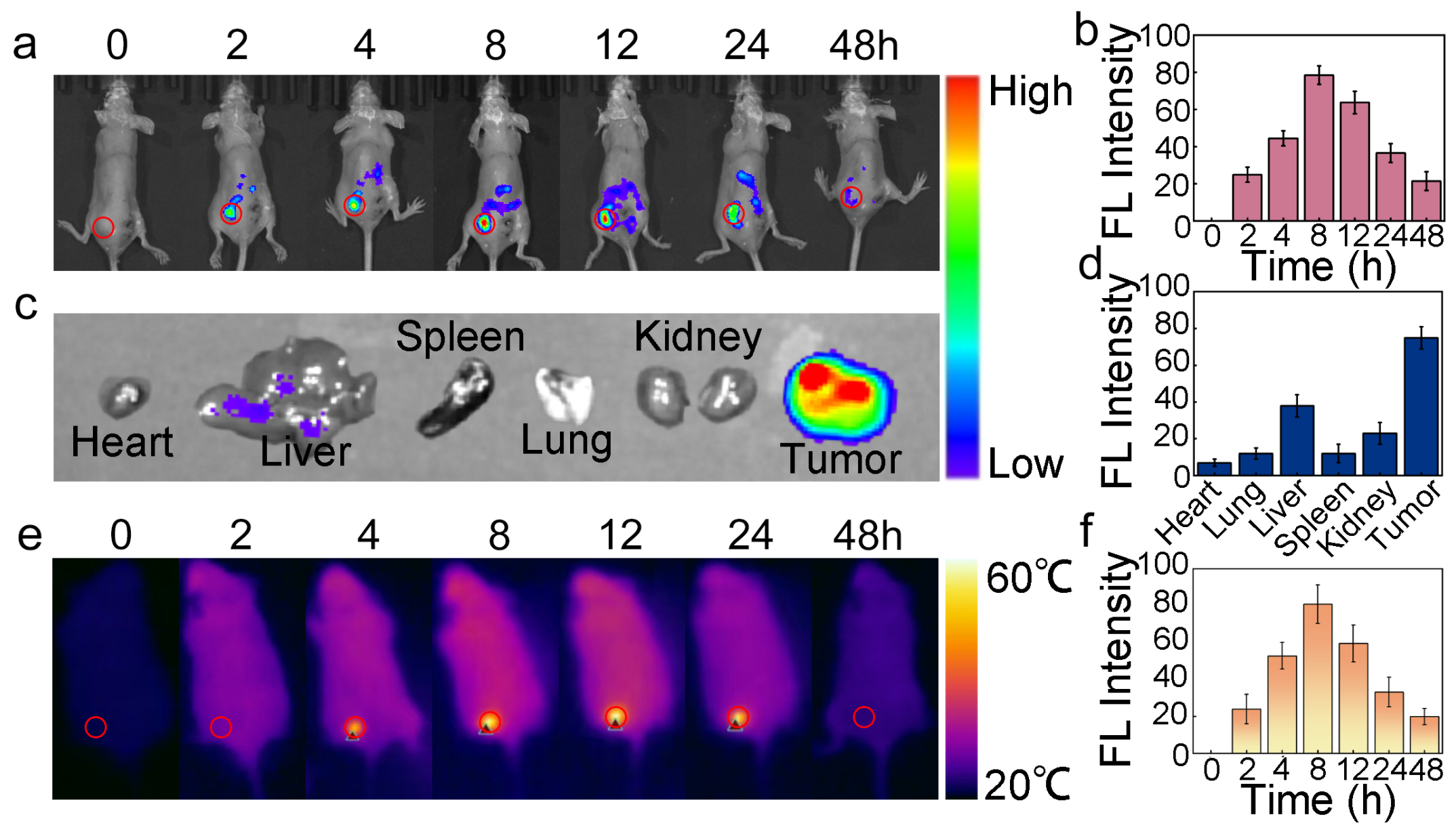


Figure S10. In vivo tumor targeting distribution and photothermal performance evaluation of HACCR in tumor-bearing mice. (a,b) In vivo fluorescence imaging and corresponding fluorescence intensity quantification at the tumor site of tumor-bearing mice at different time points after tail vein injection of HACCR. (c,d) Ex vivo fluorescence imaging and corresponding fluorescence intensity quantification of major organs and tumor tissues collected from mice at 48 h post-injection. (e,f) Infrared photothermal imaging of the tumor site under laser irradiation and corresponding temperature quantification of the tumor region at different time points after injection. Data are presented as mean ± SD (n = 3). Statistical differences were determined by one-way ANOVA.

**11 Figure S11. Hematoxylin and eosin (H&E) staining images of major organs**


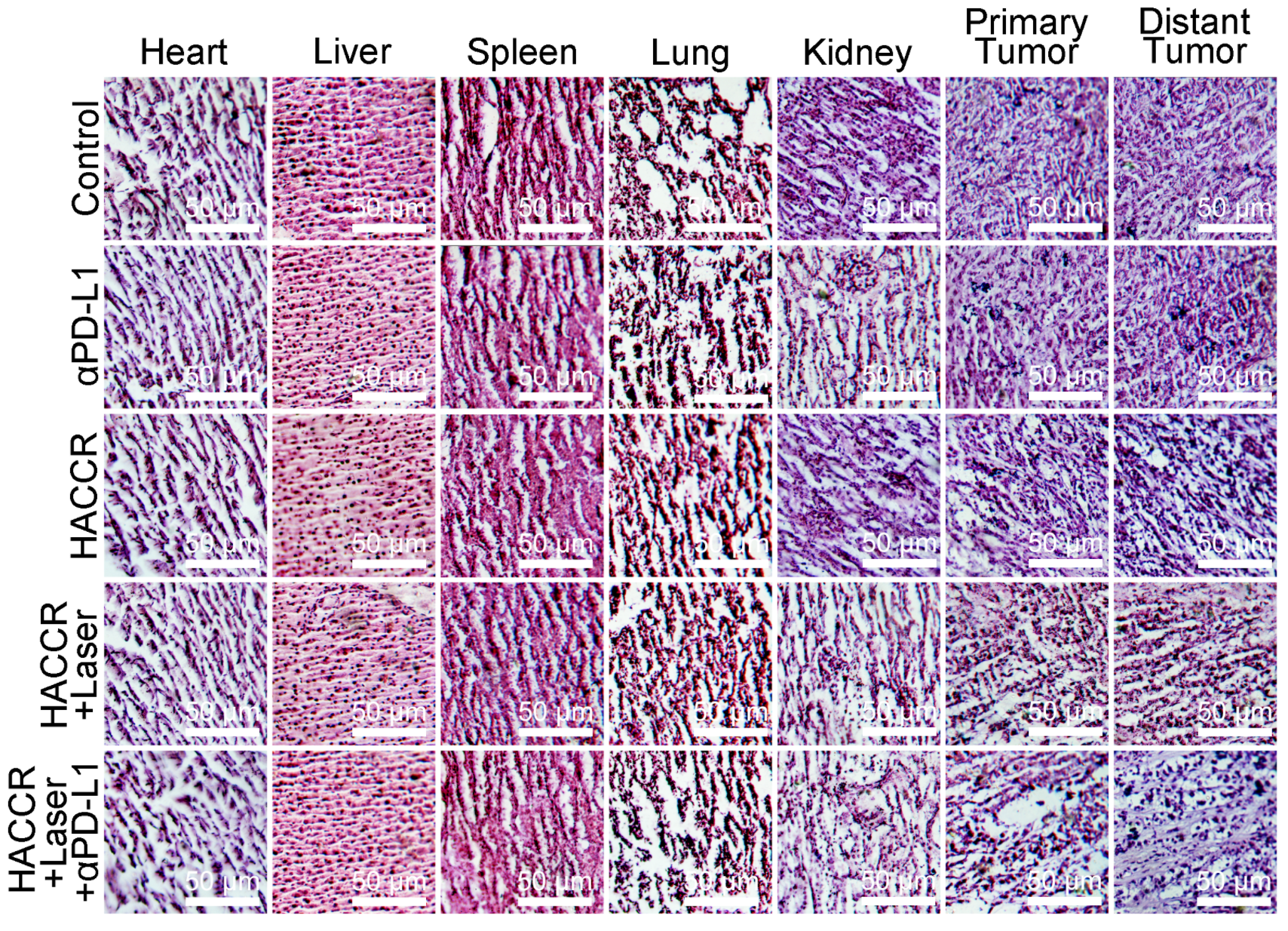


Figure S11. Hematoxylin and eosin (H&E) staining images of major organs (heart, liver, spleen, lung, kidney), primary tumor and distant tumor tissues from mice in different treatment groups, to evaluate the tissue safety and in vivo anti-tumor pathological effect of different therapeutic regimens.

**12 Figure S12. Hematoxylin and eosin (H&E) staining images of Brain**


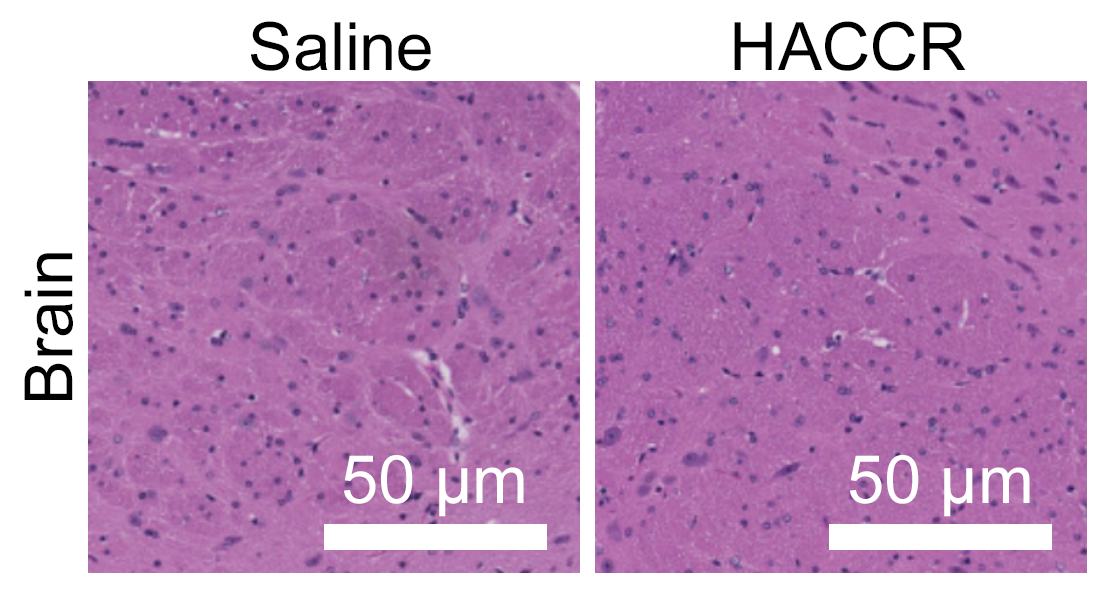


Figure S12. Hematoxylin and eosin (H&E) staining images of Brain.

**13 Figure S13. Blood biochemistry and hematology test results of mice in different treatment groups**


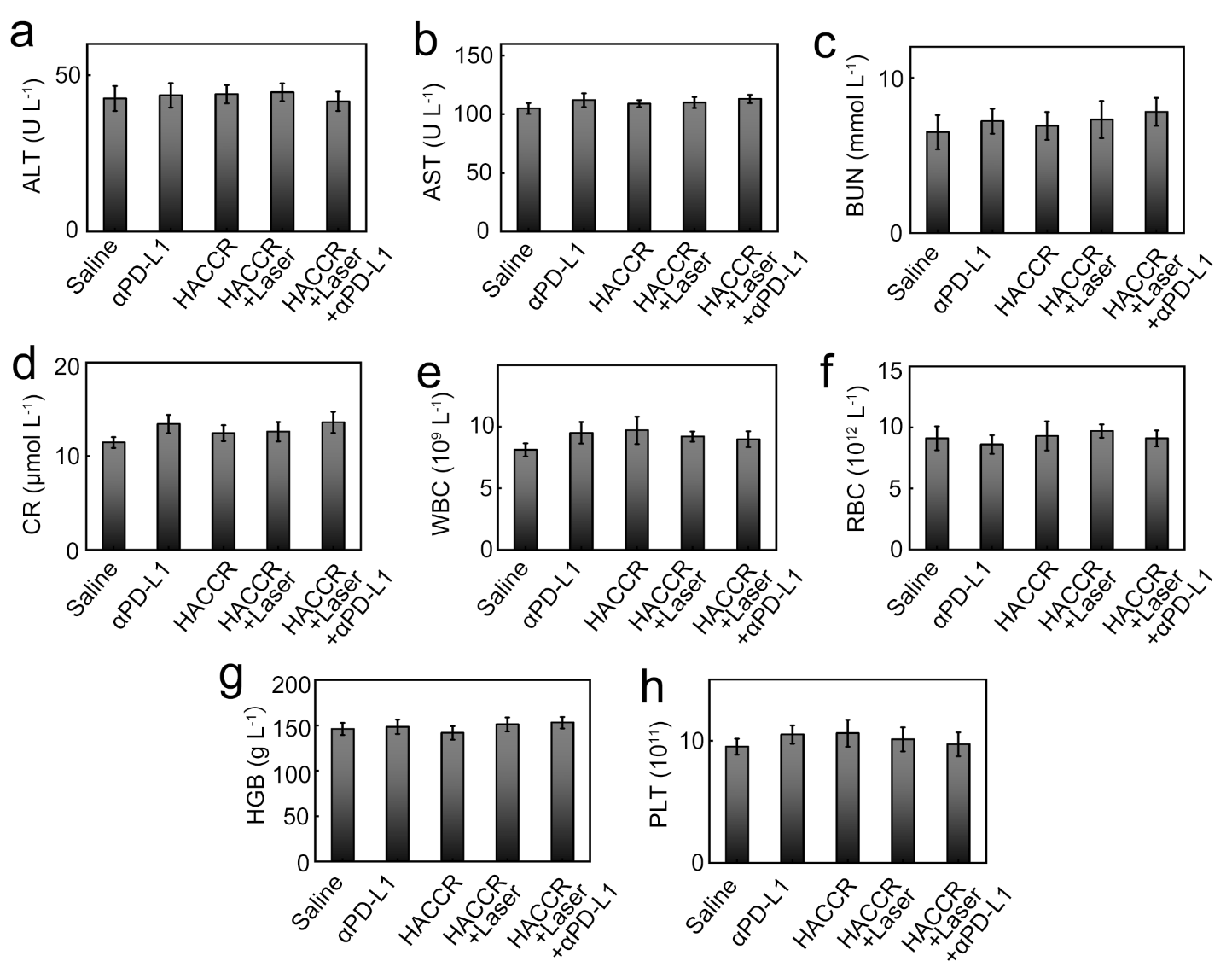


Figure S13. Blood biochemistry and hematology test results of mice in different treatment groups. (a-d) Liver function indexes: alanine aminotransferase (ALT) and aspartate aminotransferase (AST); renal function indexes: blood urea nitrogen (BUN) and creatinine (CR). (e-h) Hematology indexes: white blood cell (WBC), red blood cell (RBC), hemoglobin (HGB) and platelet (PLT), for evaluating the in vivo systemic safety of different therapeutic regimens. Data are presented as mean ± SD (n = 3). Statistical differences were determined by one-way ANOVA.

**14 Table S1. ICP-MS Detection of Copper Ion Content in Feces**

Table S1. ICP-MS Detection of Copper Ion Content in Feces

| Time (h) | Saline | HACCR (μg·mL^-1^) | Cumulative excretion (μg) | Cumulative excretion rate (%) |
| --- | --- | --- | --- | --- |
| 0 | 0.082 | 0.085 | 0 | 0 |
| 24 | 0.087 | 1.74 | 3.22 | 46.9 |
| 72 | 0.091 | 0.41 | 5.14 | 74.9 |
| 120 | 0.089 | 0.106 | 5.72 | 83.4 |
